# Dose-Dependent Preservation of Highland Barley Flour Storage Quality by *Lycium barbarum* Polysaccharide: Evidence from 240-Day Storage Monitoring and Untargeted Lipidomics

**DOI:** 10.3390/foods15203652

**Published:** 2026-10-15

**Authors:** Jidong Wu, Wenchong Shan, Miao Wang, Yong Shao, Tangwei Zhang, Ming Xiao, Yongxin She, Maojun Jin, A. M. Abd El-Aty, Jing Wang

**Affiliations:** 1 State Key Laboratory for Quality and Safety of Agro-Products, Institute of Quality Standardization & Testing Technology for Agro-Products, Chinese Academy of Agricultural Sciences, Beijing 100081, China; 2 Academy of Agriculture and Forestry Sciences, Qinghai University, Xining 810016, China; 3 National Nanfan Research Institute (Sanya), Chinese Academy of Agricultural Sciences, Sanya 572024, China; 4 Institute of Agricultural Product Quality Standard and Testing Research, Tibet Academy of Agricultural and Animal Husbandry Sciences, Lasa 850032, China; 5 Department of Pharmacology, Faculty of Veterinary Medicine, Cairo University, Giza 12211, Egypt; 6 Department of Medical Pharmacology, Faculty of Medicine, Ataturk University, Erzurum 25240, Türkiye; 7 HGF Agro, Ata Teknokent, Erzurum 25240, Türkiye

**Keywords:** highland barley flour, *Lycium barbarum* polysaccharide, storage stability, lipid oxidation, lipidomics, natural preservative

## Abstract

Highland barley (hulless barley, *Hordeum vulgare* L. var. *nudum*) flour is a nutritionally dense staple of the Qinghai–Tibet Plateau, but its high contents of unsaturated lipids and active endogenous enzymes render it highly susceptible to storage deterioration. Natural polysaccharides have been proposed as green preservatives, yet the dose-dependent effects of *Lycium barbarum* polysaccharide (LBP) on the storage quality of cereal flours and the underlying lipid-level mechanisms remain unclear. Here, highland barley flour (cv. Kunlun-15) was blended with LBP at 0.2%, 1.0% and 2.0% (*w*/*w*) stored in sealed bags at ambient temperature for 240 days, with three independently stored units per treatment; untreated flour served as the control. Storage was monitored for basic nutritional components, functional components, antioxidant activity, storage-stability indices and microbial counts, and untargeted lipidomics was performed by UPLC-MS/MS to resolve the lipid-level response. All LBP supplementations retarded the loss of nutritional and functional components, with dose-specific optima: 0.2% LBP best preserved protein, total starch, β-glucan and γ-aminobutyric acid, kept DPPH and hydroxyl radical scavenging stable, and most strongly suppressed microbial growth and the rise in peroxide value; 1.0% LBP most effectively limited moisture loss and malondialdehyde accumulation; and 2.0% LBP most strongly suppressed lipase activity, fatty acid value and the decline of polyphenols and flavonoids. Lipidomics identified 819 lipid species. Among the LBP-supplemented groups, the number of differential lipids decreased as the supplementation level increased (255, 216 and 174 for 0.2%, 1.0% and 2.0%, respectively; the control group contained 243), and LBP protected membrane phospholipids (PC, PE), storage lipids (TG) and galactolipids (MGDG) while suppressing the formation of lysophospholipids (LPC, LPE). KEGG enrichment showed that 0.2–1.0% LBP preserved glycerophospholipid and glycerolipid metabolism, whereas 2.0% LBP suppressed even the core lipid metabolic pathways. Considering the overall retention of nutritional and functional components, the stability of antioxidant activity, and the suppression of microbial growth and lipid oxidation, 0.2% LBP is recommended as the optimal supplementation level, while the lipidomic data reveal a dose-dependent, membrane-protective mechanism that supports the application of LBP as a natural preservative for whole-grain flours.

## 1. Introduction

Highland barley (hulless barley, *Hordeum vulgare* L. var. *nudum*) is the staple cereal of the Qinghai–Tibet Plateau and is prized for its high contents of β-glucan, dietary fibre, polyphenols and balanced amino acids [1]. With the rising demand for whole-grain foods, highland barley flour (HBF) is increasingly used in noodles, breads and beverages. However, HBF is one of the most unstable cereal products during storage: milling destroys the compartmentalization of the kernel and brings endogenous lipases, lipoxygenases, polyphenol oxidases and microorganisms into direct contact with their substrates, triggering hydrolytic and oxidative rancidity, protein carbonylation, phenolic degradation and microbial proliferation [2,3]. These processes generate rancid odours, discoloration and nutritional loss, severely shortening the shelf life of HBF and limiting its commercial value.

Conventional stabilization strategies for cereal flours—heat treatment, superheated steam, microwave, extrusion and modified-atmosphere packaging—can inactivate enzymes and microorganisms, but they are often energy-intensive, may alter sensory properties and nutritional quality, and are difficult to apply to small-scale production [4]. For highland barley in particular, the stabilization strategies reported so far are physical treatments applied to the grain or the flour—roasting, thermal enzyme inactivation, extrusion and superheated steam [2,3,5,6]—which act mainly by inactivating endogenous lipases and oxidases rather than by changing the storage environment of the milled flour. Adding a food-grade additive to the flour is an alternative that requires no additional thermal input and is easier to implement in small-scale production. Natural polysaccharides have therefore attracted increasing attention as green preservatives because of their antioxidant, antimicrobial and enzyme-inhibitory activities [7,8]. Polysaccharides have also been used to limit lipid oxidation and protein degradation in stored foods [9], and polysaccharide-rich flour blends can improve the storage properties of cereal flours [10]. In these studies, however, a single supplementation level was generally tested, so it remains unclear whether the protective effect increases with dose, saturates, or even reverses at higher levels. Among them, *Lycium barbarum* polysaccharide (LBP), the major bioactive fraction of wolfberry (*Lycium barbarum* L.), exhibits pronounced free-radical scavenging, chelating and antimicrobial activities [11,12]. Nevertheless, whether LBP can stabilize cereal flours during long-term storage, and how its supplementation level shapes the protective effect, has not been systematically investigated.

Lipid oxidation is widely recognized as the primary driver of quality loss in whole-grain flours [13]. Conventional indices such as fatty acid value, peroxide value and malondialdehyde content capture the bulk progress of hydrolysis and oxidation, but they cannot resolve which lipid classes and molecular species are affected. Untargeted lipidomics based on UPLC-MS/MS now allows the fate of individual membrane lipids (PC, PE), storage lipids (TG), galactolipids (MGDG), sphingolipids and their hydrolytic products (LPC, LPE) to be tracked, providing mechanistic insight into how a preservative acts at the molecular level [14]. Untargeted lipidomics has already been used to resolve storage-induced lipid remodelling in cereal flours [14,15] and to compare a stabilization treatment or a storage condition with an untreated control, as shown for nitrogen-controlled atmospheres and storage temperature in brown rice [16,17]. In all of these studies the comparison was made between storage conditions or between one treated and one untreated sample, and the lipid-level response to a graded additive dose has not been examined. Integrating conventional quality monitoring with lipidomics should therefore reveal both the macroscopic efficacy and the molecular mode of action of LBP in flour preservation.

The present work differs from the studies above in three respects. First, a graded series of LBP levels (0.2%, 1.0% and 2.0%, *w*/*w*) was tested instead of a single addition level, which makes it possible to determine whether the protective effect is dose-dependent and whether it saturates or reverses at higher doses. Second, the flour was followed for 240 days of real-time storage at ambient temperature, rather than under accelerated conditions, so that the observed trajectories correspond to a realistic shelf-life scenario. Third, untargeted lipidomics was integrated with conventional quality indices on the same samples, which links the macroscopic quality trajectory to the lipid molecular phenotype, and each of the three supplementation levels was resolved separately rather than compared only with the untreated control. In this study, HBF was supplemented with LBP at three levels (0.2%, 1.0% and 2.0%, *w*/*w*) and stored in sealed bags under ambient conditions for 240 days. The dynamic changes in basic nutritional components, functional components, antioxidant activity, storage-stability indices and microbial counts were monitored, and untargeted lipidomics was performed on samples collected at 0 and 240 days to resolve the lipid-level response. The objectives were (i) to evaluate the dose-dependent efficacy of LBP in delaying the storage deterioration of HBF, (ii) to identify the optimal supplementation level from an integrated quality and safety perspective, and (iii) to elucidate the lipid-level mechanism by which LBP protects flour quality during storage.

## 2. Materials and Methods

### 2.1. Materials and Reagents

Highland barley (cv. Kunlun-15) was harvested in Guide County, Qinghai Province, China, in 2024. *Lycium barbarum* polysaccharide (LBP) was prepared in our laboratory from sun-dried Ningqi-1 wolfberry fruits (harvested in Nuomuhong Farm, Haixi, Qinghai). Briefly, the dried fruits were ground, defatted with petroleum ether (2 mL/g, 75 °C, 2 h) and then with 80% ethanol (2 mL/g, 80 °C, 2 h), and extracted three times with water; the combined supernatants were concentrated to one quarter of the original volume, deproteinized with the Sevag reagent (2–3 cycles), precipitated with four volumes of anhydrous ethanol at 4 °C for 20 h, and freeze-dried. The total polysaccharide content of the resulting preparation was 90% (*w*/*w*), determined by the phenol–sulfuric acid method at 490 nm using glucose as the standard. A single batch of LBP was used throughout this study. Because the structural and bioactive properties of LBP depend on the raw material, the extraction procedure and the molecular weight of the preparation, it should be noted that, apart from the total polysaccharide content, the molecular weight distribution and monosaccharide composition of this preparation were not further characterized. Protein and total starch assay kits (Boxbio, Beijing, China) were used for nutritional analysis. All other reagents were of analytical grade and purchased from Sinopharm Chemical Reagent Co., Ltd. (Beijing, China). HPLC-grade methanol, acetonitrile, isopropanol, formic acid and ammonium formate were used for lipidomic analysis.

### 2.2. Sample Preparation and Storage

Clean, dried Kunlun-15 kernels were milled with a laboratory mill (LabMill, Chopin Technologies, Chopin Technologies, Villeneuve-la-Garenne, France). The flour was thoroughly blended with LBP at 0.2%, 1.0% and 2.0% (*w*/*w*) to prepare treatment groups TG1, TG2 and TG3, respectively; flour without LBP served as the control (CK). Each group was sealed in self-sealing polyethylene bags and stored at ambient temperature (average 11 °C, average relative humidity ca. 45%) for 240 days. For each group, three independent storage units (separately sealed bags, each containing an equal portion of the same blended batch) were prepared and stored in parallel, and each unit was opened once and sampled destructively. The three replicates reported at every storage time are therefore independently stored samples (separate storage units) rather than analytical replicates of a single sample. Samples were withdrawn at 0, 60, 120, 180 and 240 days for quality analysis; samples for lipidomics were collected at 0 and 240 days with six biological replicates each and stored at −80 °C.

### 2.3. Basic Nutritional Components

Moisture content was determined by the direct drying method according to GB 5009.3-2016 [18]; crude fat was determined by acid hydrolysis according to GB 5009.6-2016 [19]; and protein and total starch contents were quantified with Boxbio assay kits (Beijing Boxbio Science & Technology Co., Ltd., Beijing, China).

### 2.4. Functional Components and Antioxidant Activity

β-Glucan content was determined with a Boxbio assay kit. Total phenolic content was measured by the Folin–Ciocalteu colorimetric method [20]; total flavonoid content was determined according to the method of Yang et al. [21]; and γ-aminobutyric acid (GABA) content was determined by the Berthelot colorimetric method [22]. The DPPH free radical scavenging rate was determined according to the method of Chen et al. [23] and Gu et al. [24]. The determination of the ABTS radical scavenging rate was based on the method of Zhang et al. [25]. The determination of the hydroxyl radical scavenging rate is based on the method of Sun et al. [26].

### 2.5. Storage-Stability Indices and Microbial Enumeration

Lipase activity was determined according to GB/T 5523-2008 [27]; fatty acid value was determined by manual titration according to GB/T 5510-2024 [28]; malondialdehyde (MDA) content was quantified with a Boxbio assay kit; peroxide value was determined according to the method of Zheng et al. [5]; and peroxidase activity was measured by the guaiacol oxidation method. Total plate count and mould count were determined according to GB 4789.2-2022 and GB 4789.15-2016 [29,30], respectively.

### 2.6. Untargeted Lipidomics

Flour samples (100 mg) were extracted with 800 µL of pre-cooled (−20 °C) MTBE/methanol (3:1, *v*/*v*) containing internal standards. After grinding, sonication and incubation at −20 °C for 2 h, 500 µL of water/methanol (3:1, *v*/*v*) was added; the mixture was vortexed and centrifuged. The supernatant (600 µL) was dried under vacuum, reconstituted in 400 µL of reconstitution solution, and analysed by UPLC-MS/MS on a Q Exactive Focus instrument (Thermo Fisher Scientific, Waltham, MA, USA) equipped with a Waters CSH C18 column (1.7 µm, 2.1 × 100 mm) at 55 °C. The mobile phase for positive mode consisted of (A) 60% acetonitrile in water containing 10 mM ammonium formate and 0.1% formic acid and (B) 90% isopropanol/10% acetonitrile containing 10 mM ammonium formate and 0.1% formic acid; formic acid was omitted in negative mode. Gradient elution was performed at 0.4 mL/min. Electrospray ionization was run in positive/negative switching mode. Raw data were processed with LipidSearch 4.0 for peak picking, identification and quantification and annotated against the KEGG, HMDB and LIPID MAPS databases. Data were preprocessed with metaX; trend clustering was performed with Mfuzz; and KEGG pathway enrichment of differential lipids was performed using the KEGG database. Before analysis, lipid features were filtered (missing values in >50% of the QC samples or >80% of the experimental samples were removed), imputed using the KNN algorithm, normalized by probabilistic quotient normalization (PQN), and corrected for batch effects by QC-RLSC, and features with a coefficient of variation >30% across QC samples were discarded. Quality control (QC) samples were prepared by pooling equal volumes of all test samples.

### 2.7. Statistical Analysis

All experiments were performed in triplicate unless otherwise stated; as described in Section 2.2, the three replicates were independently stored samples. Data are expressed as mean ± standard deviation (*n* = 3 independently stored samples). Error bars in all figures represent standard deviation. For the conventional quality and microbial indices, the four groups (CK, TG1, TG2 and TG3) were compared separately at each storage time by one-way analysis of variance (ANOVA) followed by Duncan’s multiple range test (SPSS 26.0, IBM, Armonk, NY, USA); *p* < 0.05 was considered statistically significant. Data from different storage times were never pooled in a single comparison, and the effects of LBP level and storage time were therefore not evaluated jointly. Correlation analysis and figure preparation were performed with Origin Pro 2025. Multivariate statistics (PCA and OPLS-DA) were performed with the metaX pipeline; differential metabolites (referred to hereafter as differential lipids) were defined as those satisfying all three of the following criteria simultaneously: (i) VIP ≥ 1 in the PLS-DA model, (ii) fold change ≥1.2 or ≤0.83, and (iii) *p* < 0.05 from a two-tailed Student’s *t*-test.

## 3. Results

### 3.1. Baseline Comparability of the Treatment Groups

The indices measured at day 0 establish the baseline against which the changes during storage were assessed (Table 1). Because the four groups were prepared by blending sub-portions of the same batch of flour, the day-0 values are not numerically identical, and the four groups were therefore compared by one-way ANOVA followed by Duncan’s test to confirm that no significant differences existed at the start of storage. No significant differences (*p* > 0.05) were found among the four groups for any of the indices in Table 1, indicating that the small numerical differences among them reflect measurement variation rather than a systematic difference in the starting material. The day-0 values can therefore be used as a common baseline for evaluating the subsequent storage changes.

### 3.2. The Results of Basic Nutritional Components

**Figure 1:**
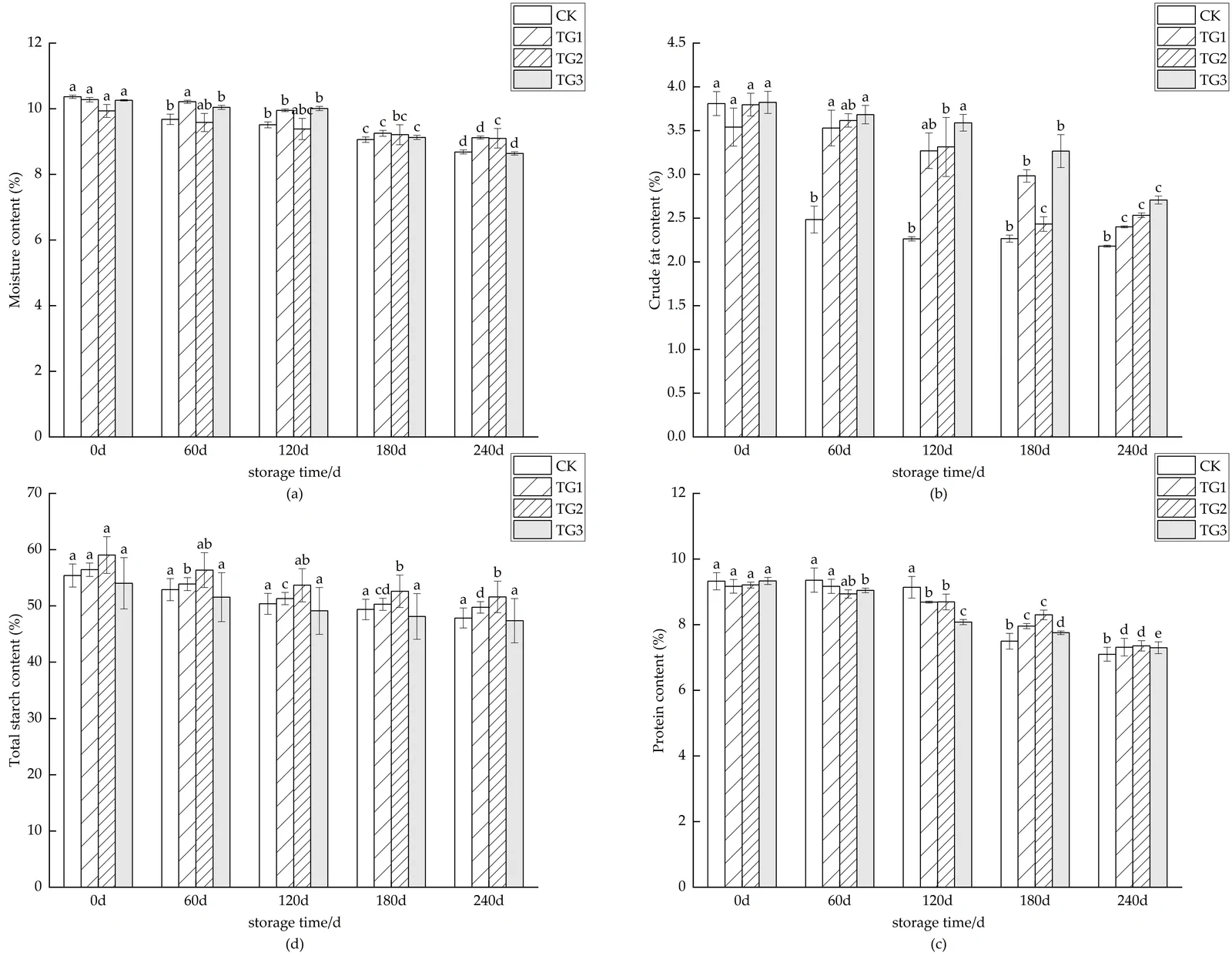
Changes in the basic nutritional components of highland barley flour during 240 days of storage: (**a**) moisture content, (**b**) crude fat content, (**c**) total protein content and (**d**) total starch content. (CK, control flour without LBP; TG1, TG2 and TG3, flour supplemented with 0.2%, 1.0% and 2.0% (*w*/*w*) LBP, respectively). Different lowercase letters indicate significant differences among the storage times within each group (Duncan’s multiple-range test, *p* < 0.05); the letters compare the sampling points of a single curve and are not comparable between curves.

The moisture content of all groups declined gradually during storage (Figure 1a), from 10.36% to 8.68% in CK and from 10.23% to 9.10% in TG2, reflecting the slow evaporation of free water through the packaging [3]. The total decline over the 240-day period was smallest in TG2 (1.13 percentage points), and this difference from CK was statistically significant only at 240 days, indicating that 1.0% LBP retained moisture most effectively over the storage period, presumably through hydrogen bonding between the hydroxyl groups of the polysaccharide and water molecules [6].

Crude fat content decreased continuously in all groups (Figure 1b), from 3.81% to 2.18% in CK, but only from 3.82% to 2.71% in TG3. The slower decline in TG3 suggests that a higher LBP level forms a protective film around fat globules, reducing the contact between lipase and its substrate [31].

Protein content declined in all groups (Figure 1c), from 9.32% to 7.10% in CK, from 9.17% to 7.31% in TG1, from 9.21% to 7.35% in TG2 and from 9.33% to 7.29% in TG3. The loss was smallest in TG1 (1.86 percentage points), followed by TG2 (1.86 percentage points), TG3 (2.04 percentage points) and CK (2.22 percentage points). The smaller losses in the treated groups are consistent with LBP adsorbing onto protein surfaces and forming gel networks that shield proteins from microbial proteases and endogenous proteolysis [32,33].

Total starch content (Figure 1d) decreased from 55.41% to 47.85% in CK (a 13.6% loss), but only from 56.45% to 49.75% in TG1 (11.9%), indicating that 0.2% LBP slightly inhibited amylase activity and reduced starch degradation. Overall, LBP at 0.2% and 1.0% effectively slowed the decline of the basic nutritional components, whereas 2.0% LBP most strongly protected the fat fraction.

### 3.3. The Results of Functional Components and Antioxidant Activity

**Figure 2:**
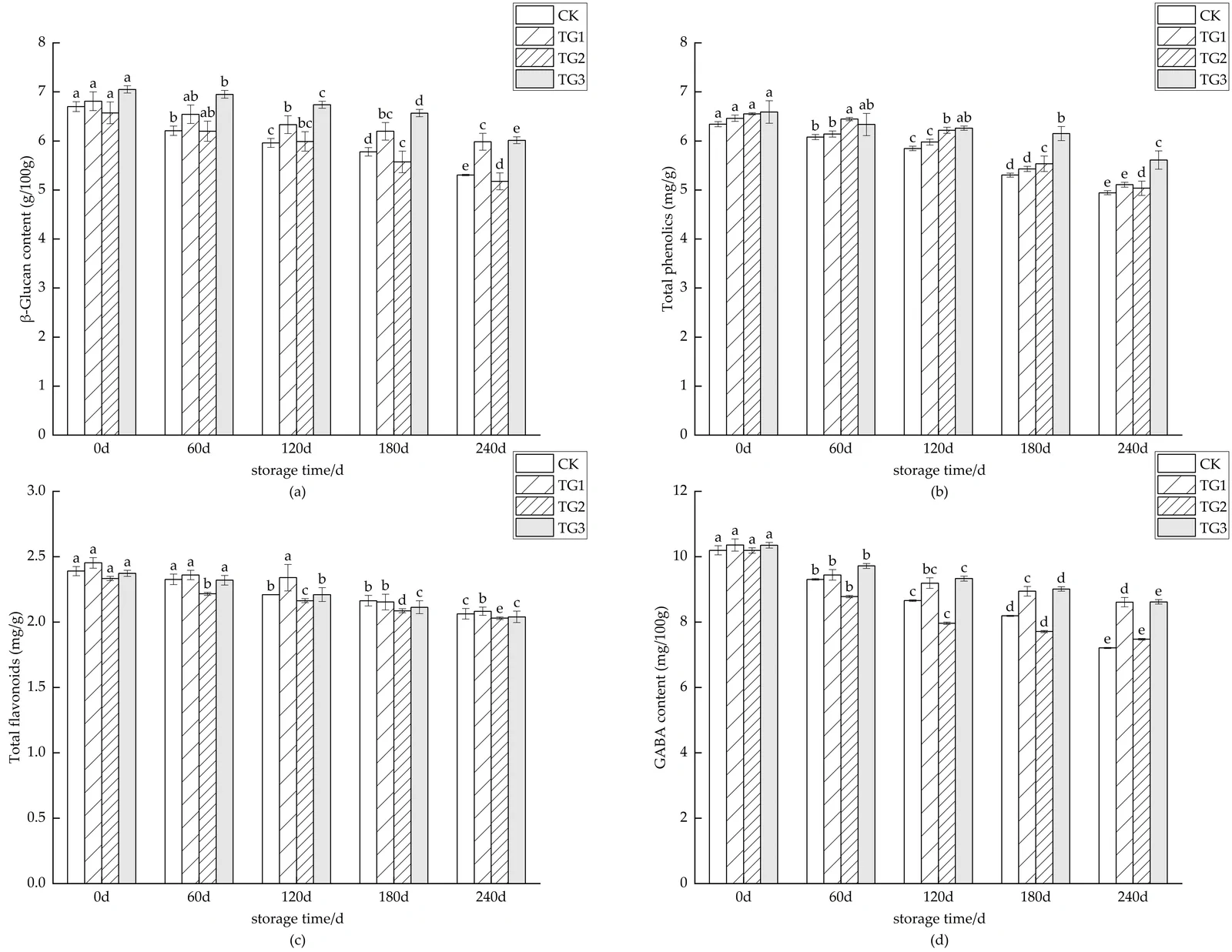
Changes in β-glucan content (**a**), total phenolic content (**b**), total flavonoid content (**c**) and γ-aminobutyric acid (GABA) content (**d**) of highland barley flour during storage (CK, control flour without LBP; TG1, TG2 and TG3, flour supplemented with 0.2%, 1.0% and 2.0% (*w*/*w*) LBP, respectively). Different lowercase letters indicate significant differences among the storage times within each group (Duncan’s multiple-range test, *p* < 0.05); the letters compare the sampling points of a single curve and are not comparable between curves.

β-Glucan content declined in all groups (Figure 2a), and the slowest decline occurred in TG1 (6.81 → 5.98 g/100 g versus 6.70 → 5.30 g/100 g in CK), consistent with the inhibition of glucanase by 0.2% LBP and reduced microbial consumption of β-glucan [34,35].

Total phenolics and total flavonoids decreased in all groups (Figure 2b,c), with the slowest decline in TG3 (total phenolics 6.59 → 5.61 mg/g; total flavonoids 2.37 → 2.09 mg/g), where the higher polysaccharide level most effectively inhibited polyphenol oxidase and shielded phenolics from oxygen via complex formation [36].

GABA content declined in all groups (Figure 2d), from 10.20 to 7.21 mg/100 g in CK versus 10.36 to 8.61 mg/100 g in TG1; the slower decline in TG1 is consistent with inhibition of transaminase activity by the polysaccharide [37,38,39].

**Figure 3:**
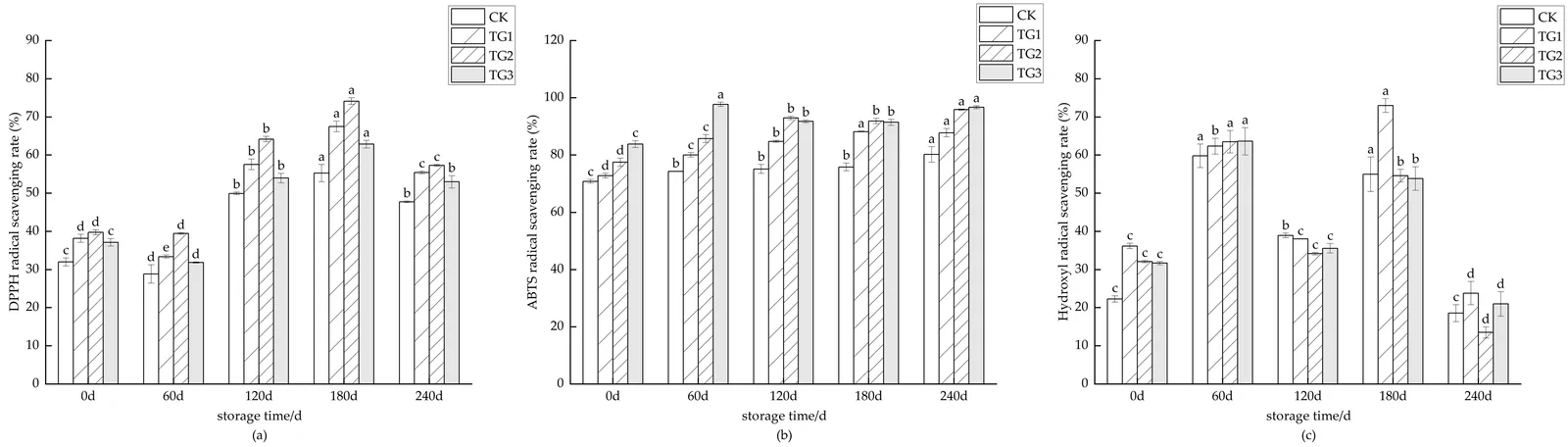
Changes in DPPH radical scavenging rate (**a**), ABTS radical scavenging rate (**b**) and hydroxyl radical scavenging rate (**c**) of highland barley flour during storage (CK, control flour without LBP; TG1, TG2 and TG3, flour supplemented with 0.2%, 1.0% and 2.0% (*w*/*w*) LBP, respectively). Different lowercase letters indicate significant differences among the storage times within each group (Duncan’s multiple-range test, *p* < 0.05); the letters compare the sampling points of a single curve and are not comparable between curves.

Antioxidant activities showed dose- and time-dependent dynamics [40]. DPPH radical scavenging followed a decline–rise–decline pattern in all groups, peaking at 180 days (e.g., 55.30% in CK), with the most stable profile in TG1 (Figure 3a). ABTS radical scavenging increased throughout storage in all groups (Figure 3b), most strongly in TG3 (83.87% → 96.72%), consistent with the progressive degradation of macromolecular LBP into smaller fragments with higher radical-scavenging activity [41,42]. Hydroxyl radical scavenging fluctuated with a net decline (Figure 3c), with TG1 maintaining the highest overall level. Together, 0.2% LBP preserved the most stable antioxidant profile, whereas higher levels produced transiently higher ABTS activity but larger fluctuations.

### 3.4. The Results of Storage-Stability Indices and Microbial Counts

**Figure 4:**
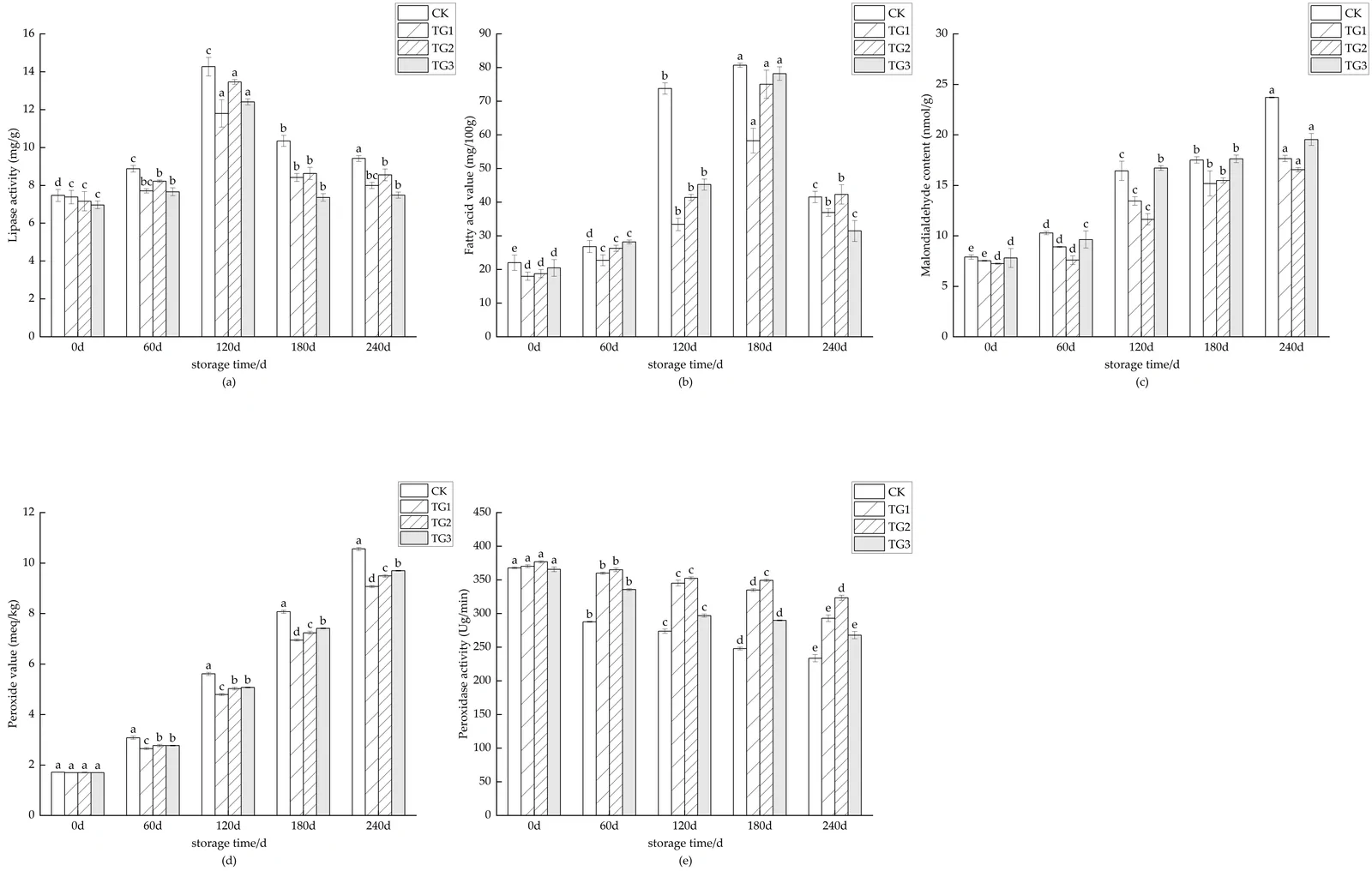
Changes in lipase activity (**a**), fatty acid value (**b**), malondialdehyde (MDA) content (**c**), peroxide value (**d**) and peroxidase activity (**e**) of highland barley flour during storage (CK, control flour without LBP; TG1, TG2 and TG3, flour supplemented with 0.2%, 1.0% and 2.0% (*w*/*w*) LBP, respectively). Different lowercase letters indicate significant differences among the storage times within each group (Duncan’s multiple-range test, *p* < 0.05); the letters compare the sampling points of a single curve and are not comparable between curves.

Lipase activity increased to a maximum at 120 days and then declined in all groups (Figure 4a); TG3 consistently showed the lowest activity (e.g., 14.27 mg/g at 120 days in CK), indicating that 2.0% LBP most effectively suppressed microbial enzyme production and activation of endogenous lipase. Fatty acid value followed a parallel rise–fall pattern with a peak at 180 days (80.67 mg/100 g in CK) and was lowest in TG3 throughout (Figure 4b).

MDA content increased continuously in all groups (Figure 4c), from 7.90 to 23.70 nmol/g in CK versus 7.24 to 16.55 nmol/g in TG2. This is because with the extension of storage time, the degree of fat oxidation is also deepening, and the amount of malondialdehyde generated will continue to increase [9], showing that 1.0% LBP most effectively slowed lipid peroxidation. Peroxide value increased in all groups, most slowly in TG1 (Figure 4d). Peroxidase activity declined linearly in all groups (Figure 4e), most slowly in TG2 (367.81 → 323.51 U/g·min, a 12.0% decrease) and most rapidly in CK (376.75 → 233.62 U/g·min, a 38.0% decrease), with intermediate decreases in TG1 (20.8%) and TG3 (26.7%).

**Figure 5:**
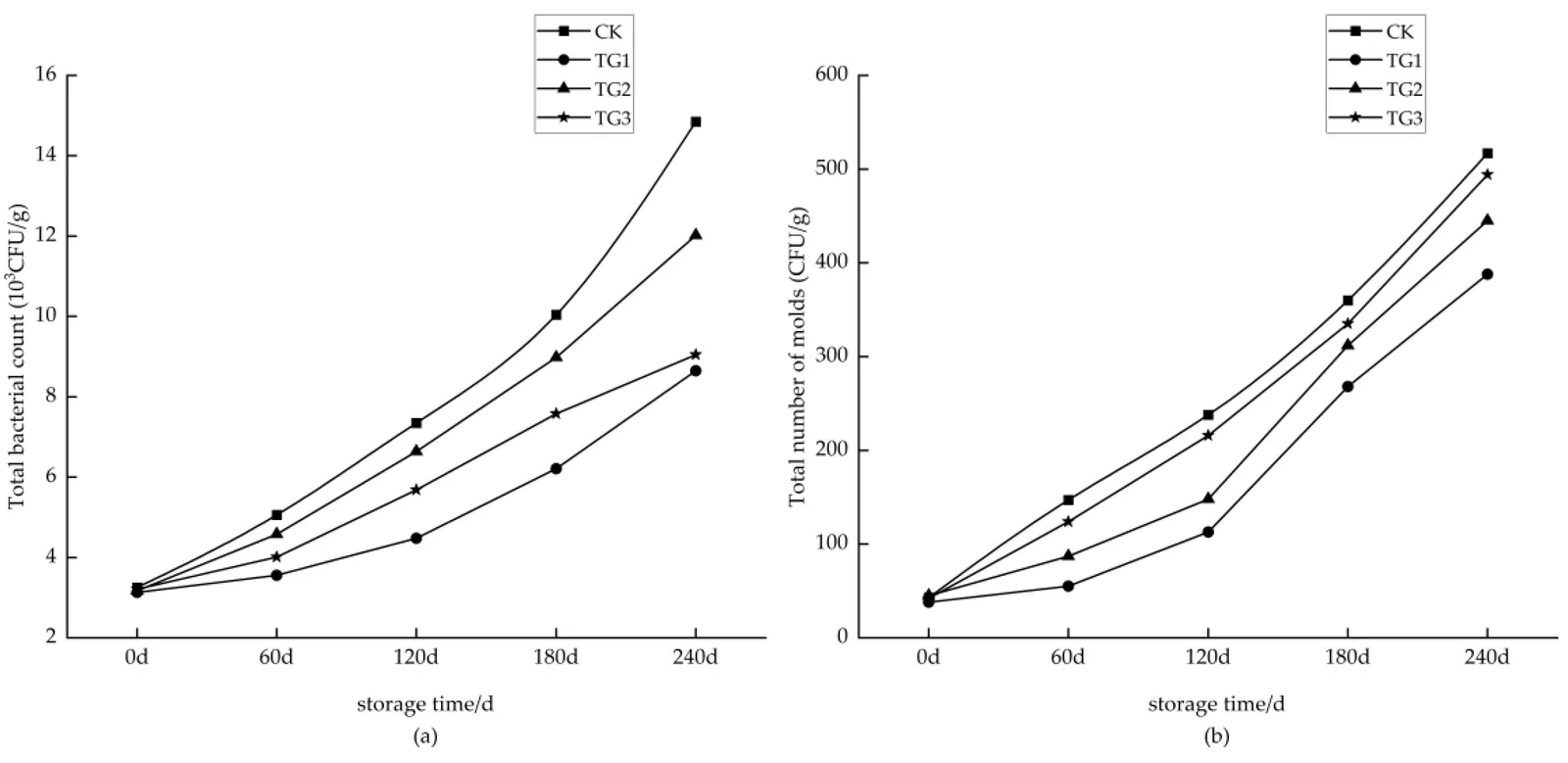
Changes in total plate count (**a**) and mould count (**b**) of highland barley flour during storage (CK, control flour without LBP; TG1, TG2 and TG3, flour supplemented with 0.2%, 1.0% and 2.0% (*w*/*w*) LBP, respectively).

Total plate count and mould count increased throughout storage (Figure 5). All LBP-supplemented groups suppressed the growth of both bacteria and moulds relative to CK; the highest counts were recorded in the control group throughout storage. The suppressive effect nevertheless weakened as the supplementation level increased: counts in TG3 increased faster than in TG1, suggesting that at high levels the added polysaccharide can also serve as a carbon source for microorganisms [43,44]. TG1 showed the lowest total plate count and mould count, indicating that 0.2% LBP provided the best microbiological control.

### 3.5. Correlation Analysis

**Figure 6:**
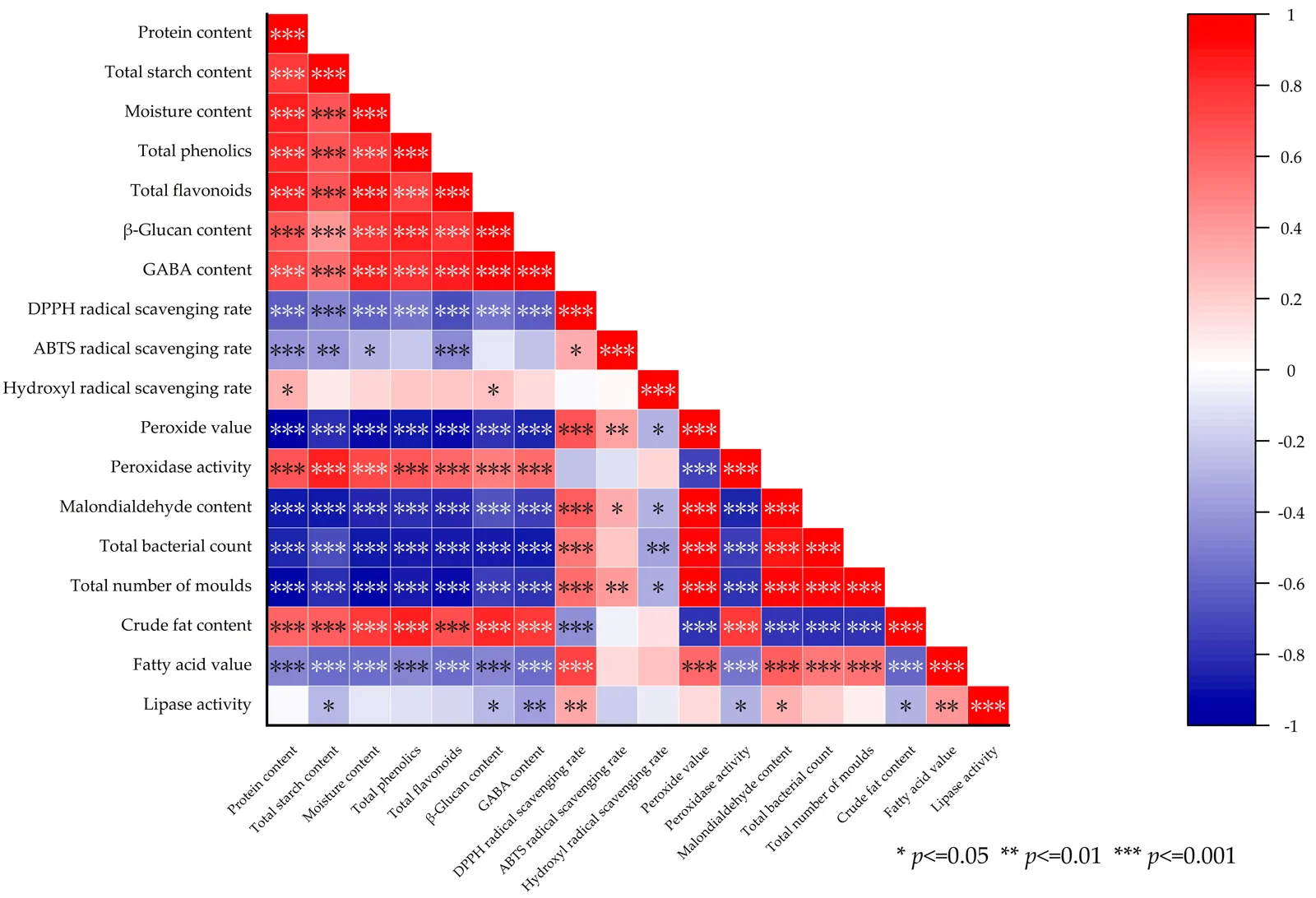
Pearson correlation analysis of 18 quality indices of highland barley flour during 240-day storage. The four panels correspond to CK (control flour without LBP), TG1, TG2 and TG3 (flour supplemented with 0.2%, 1.0% and 2.0% (*w*/*w*) LBP, respectively). Rows and columns represent the same 18 indices; the diagonal is omitted. Each circle gives the Pearson correlation coefficient r between the corresponding pair of indices, with circle size and colour depth proportional to |r|; red indicates a positive correlation and blue a negative correlation. Asterisks denote the significance of the correlation (* *p* < = 0.05, ** *p* < = 0.01, *** *p* < = 0.001).

Pearson correlation analysis of 18 indices over the 240-day storage period (Figure 6) showed that, in CK, the basic nutritional components were significantly and positively intercorrelated, and MDA content correlated positively with total plate and mould counts, linking microbial proliferation to lipid oxidation [45]. In TG1, functional components became negatively correlated with MDA content and microbial indices, indicating that the polysaccharide allowed functional components to exert antioxidant and antimicrobial effects. In TG2 and TG3, the antagonism between functional components and oxidation indices became progressively clearer, with TG3 showing the strongest negative correlation between functional components and MDA. Peroxide value correlated positively with mould count in the middle and late stages, MDA correlated negatively with peroxidase activity throughout, and ABTS scavenging correlated positively with total phenolics in the late stage, confirming that phenolics remained functionally relevant during prolonged storage.

### 3.6. Lipidomic Characterization of Stored Flour

#### 3.6.1. Global Separation and Lipid Inventory

**Figure 7:**
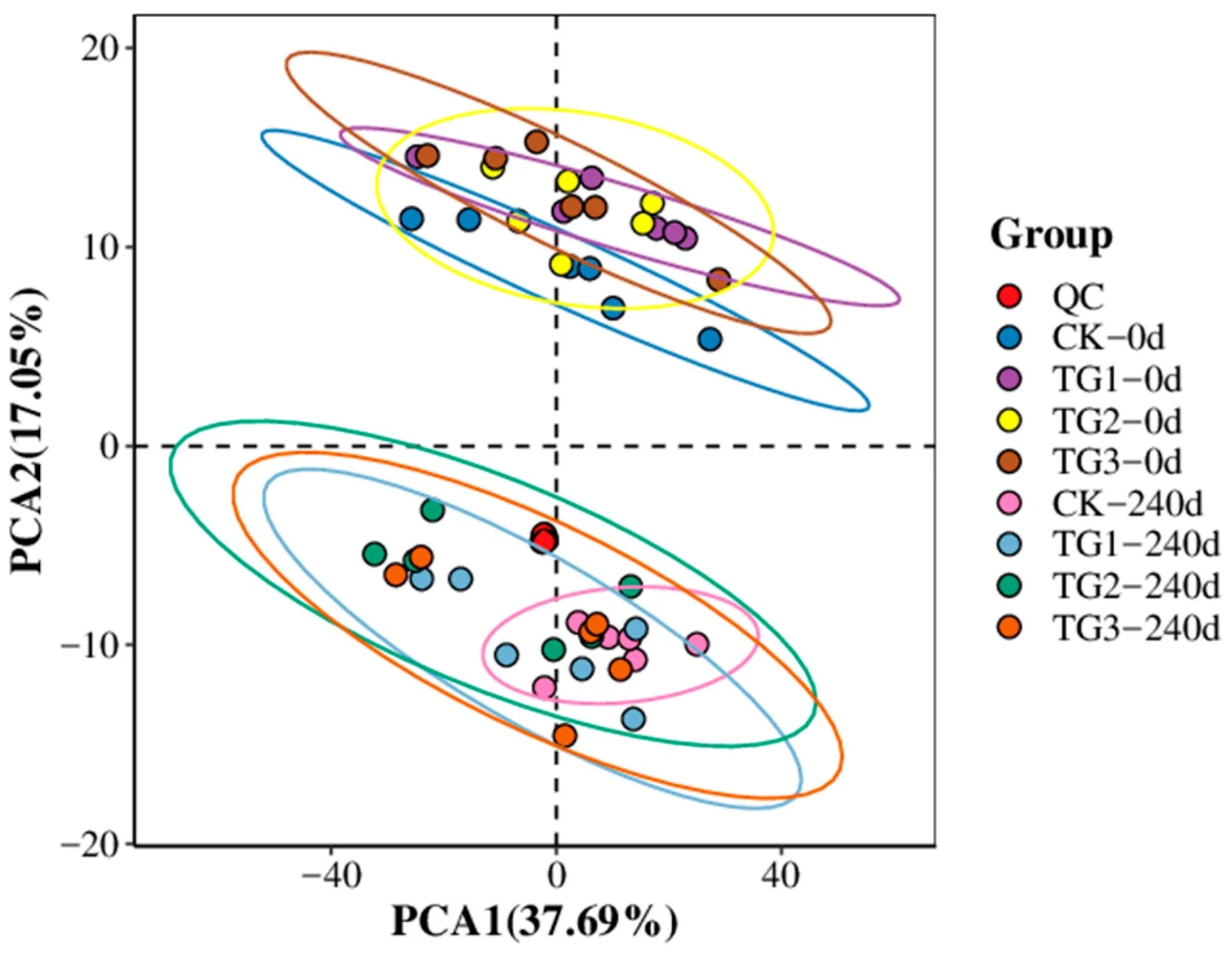
PCA score plot of QC samples and all highland barley flour samples (CK–0 d, TG1/2/3–0 d: control and treatment groups at day 0; CK–240 d, TG1/2/3–240 d: control and treatment groups at day 240; the same below) (CK, control flour without LBP; TG1, TG2 and TG3, flour supplemented with 0.2%, 1.0% and 2.0% (*w*/*w*) LBP, respectively).

PCA of all samples and QC pools (Figure 7) showed tight clustering of QC samples, confirming good analytical repeatability. All samples collected at day 0 separated completely from those collected at day 240 along PC2, indicating that storage time was the dominant factor shaping the lipid metabolic phenotype, whereas the treatment groups diverged from CK along PC1 after 240 days, showing that LBP modified the lipidomic response in a dose-dependent manner.

**Figure 8:**
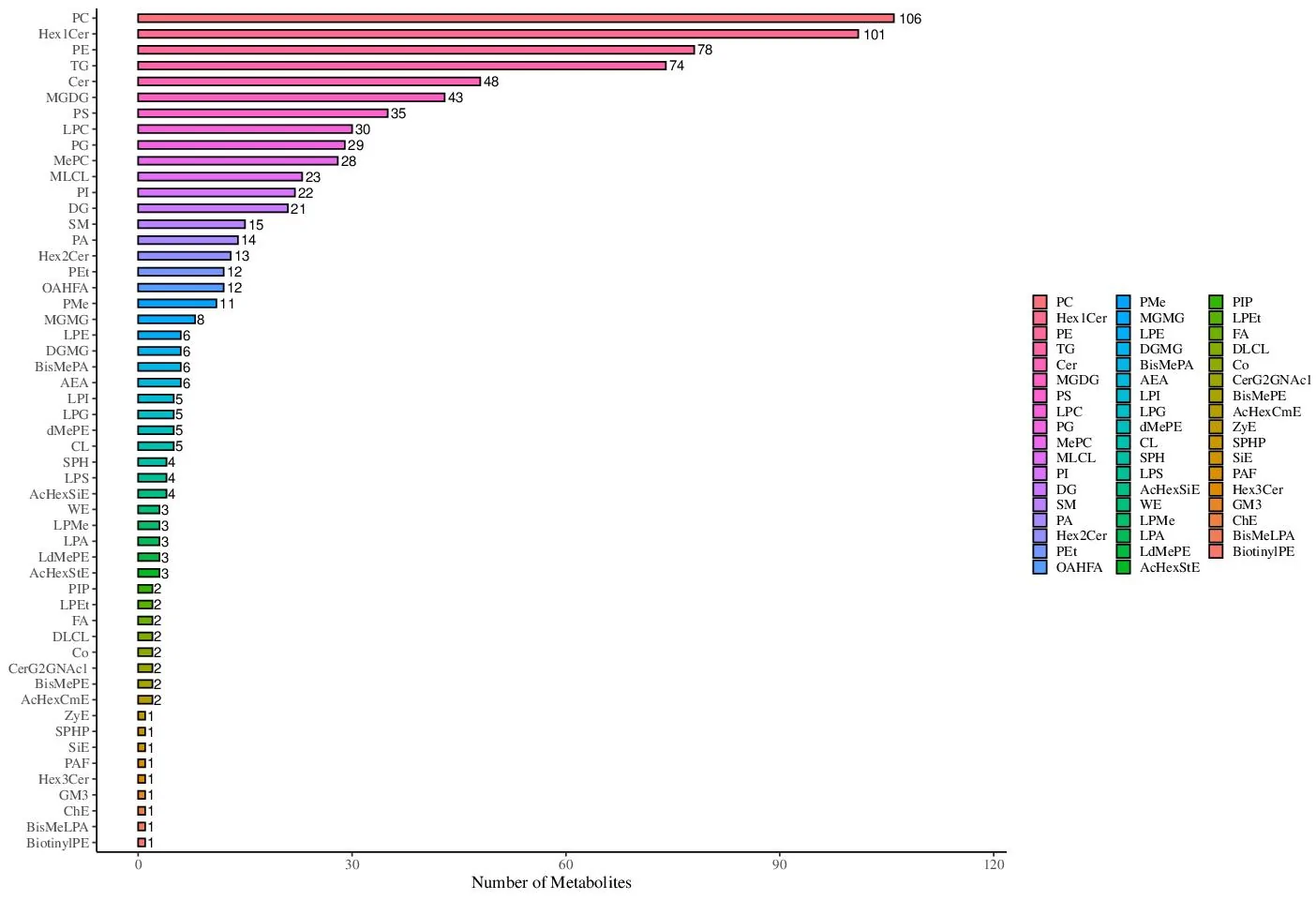
Lipid categories identified in the overall sample set.

**Figure 9:**
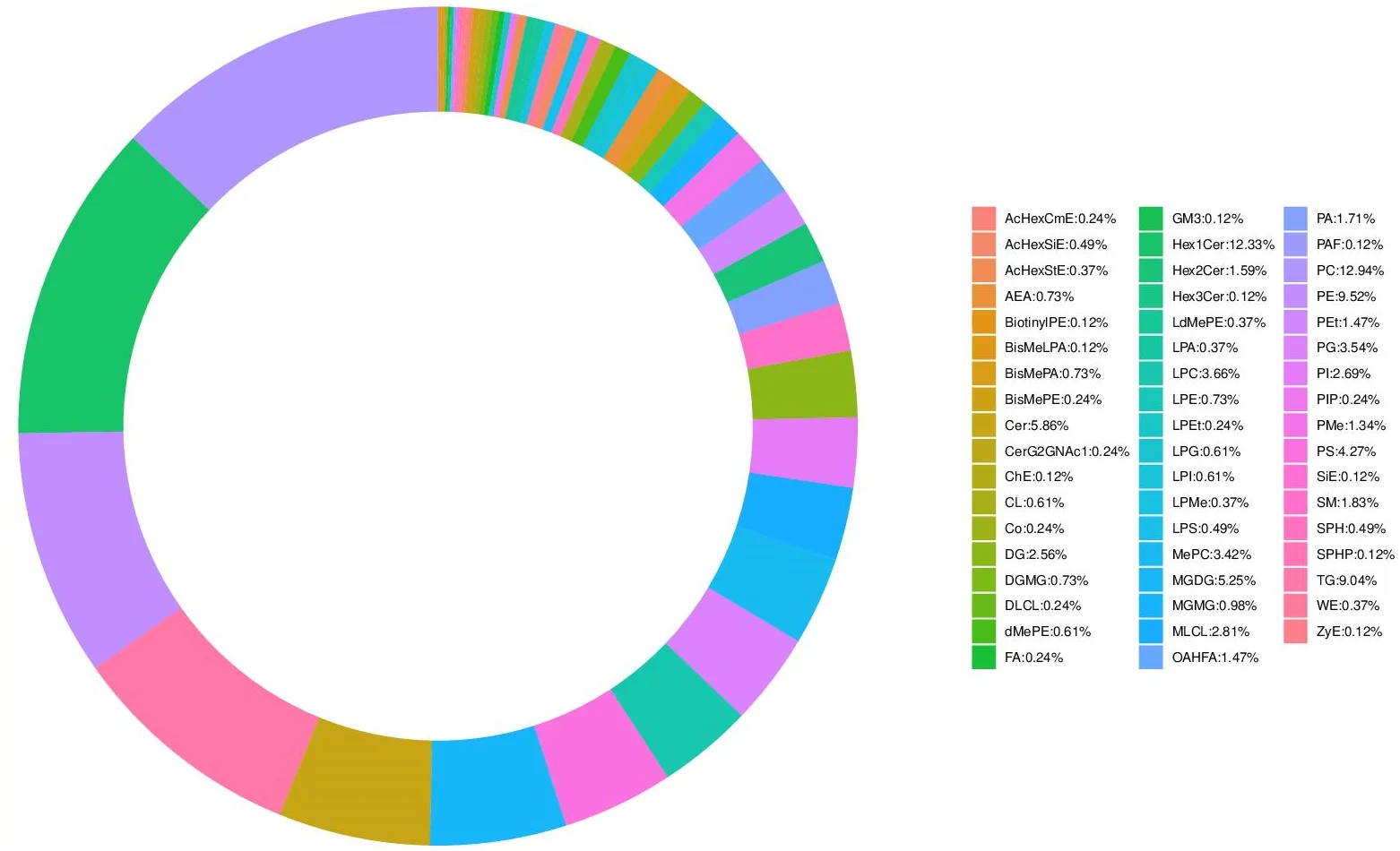
Overall lipid composition of the samples (number and percentage of lipid species by class).

In total, 819 lipid species were identified, covering the major plant lipid categories (Figure 8): glycerophospholipids (GPs), glycerolipids (GLs), sphingolipids (SPs), fatty acyls (FAs) and sterol lipids (STs). Glycerophospholipids were the most abundant class, with phosphatidylcholine (PC, 106 species, 12.94%), hexosylceramide (Hex1Cer, 101 species, 12.33%) and phosphatidylethanolamine (PE, 78 species, 9.52%) being the most numerous; triacylglycerols (TG, 74 species, 9.04%) represented the main storage lipids, and monogalactosyldiacylglycerol (MGDG, 43 species, 5.25%), ceramides (Cer, 48 species, 5.86%) and lysophosphatidylcholine (LPC, 35 species, 4.27%) were also detected (Figure 9).

#### 3.6.2. Group-Wise PCA and OPLS-DA

**Figure 10:**
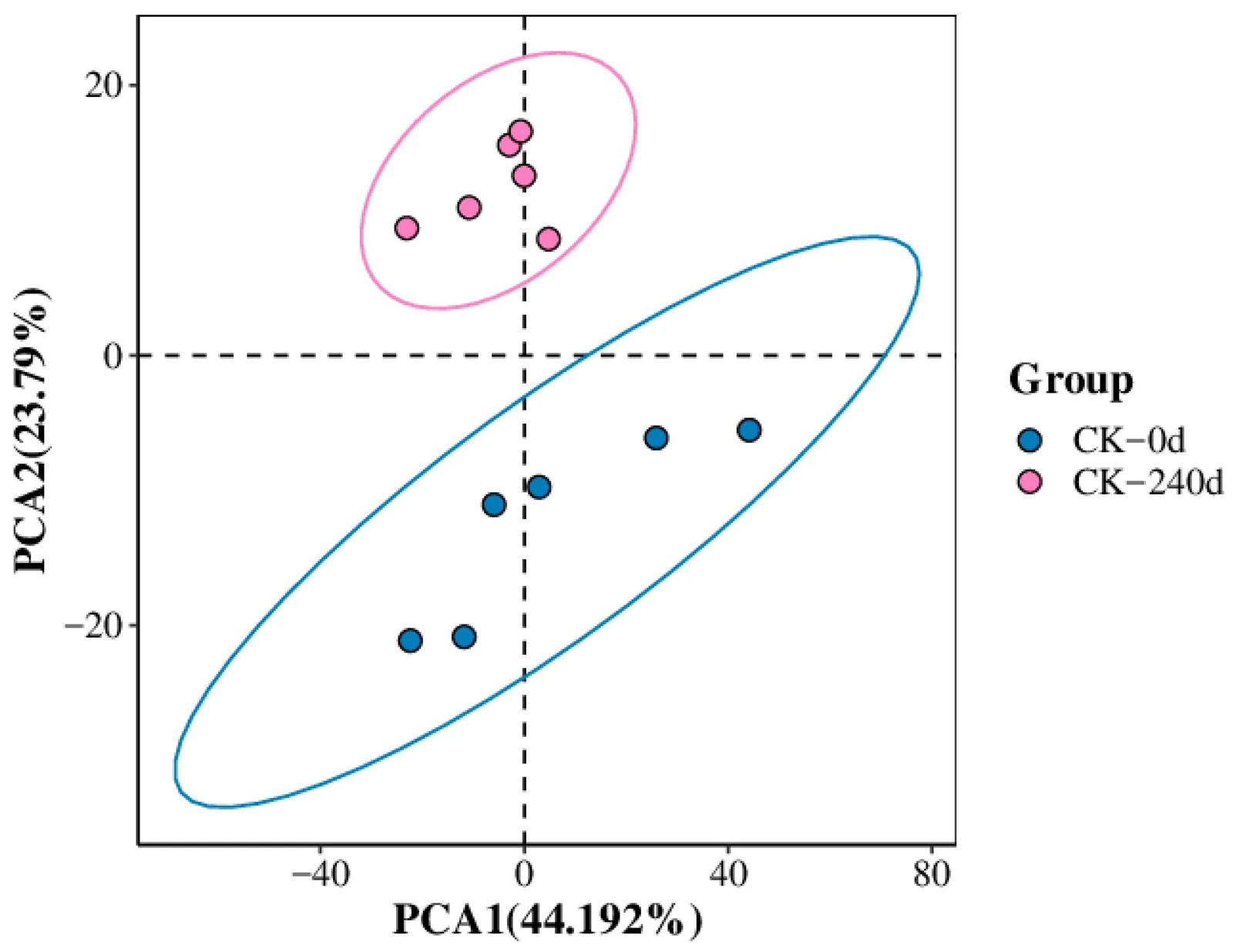
PCA score plot of the control group before and after storage (CK, control flour without LBP).

**Figure 11:**
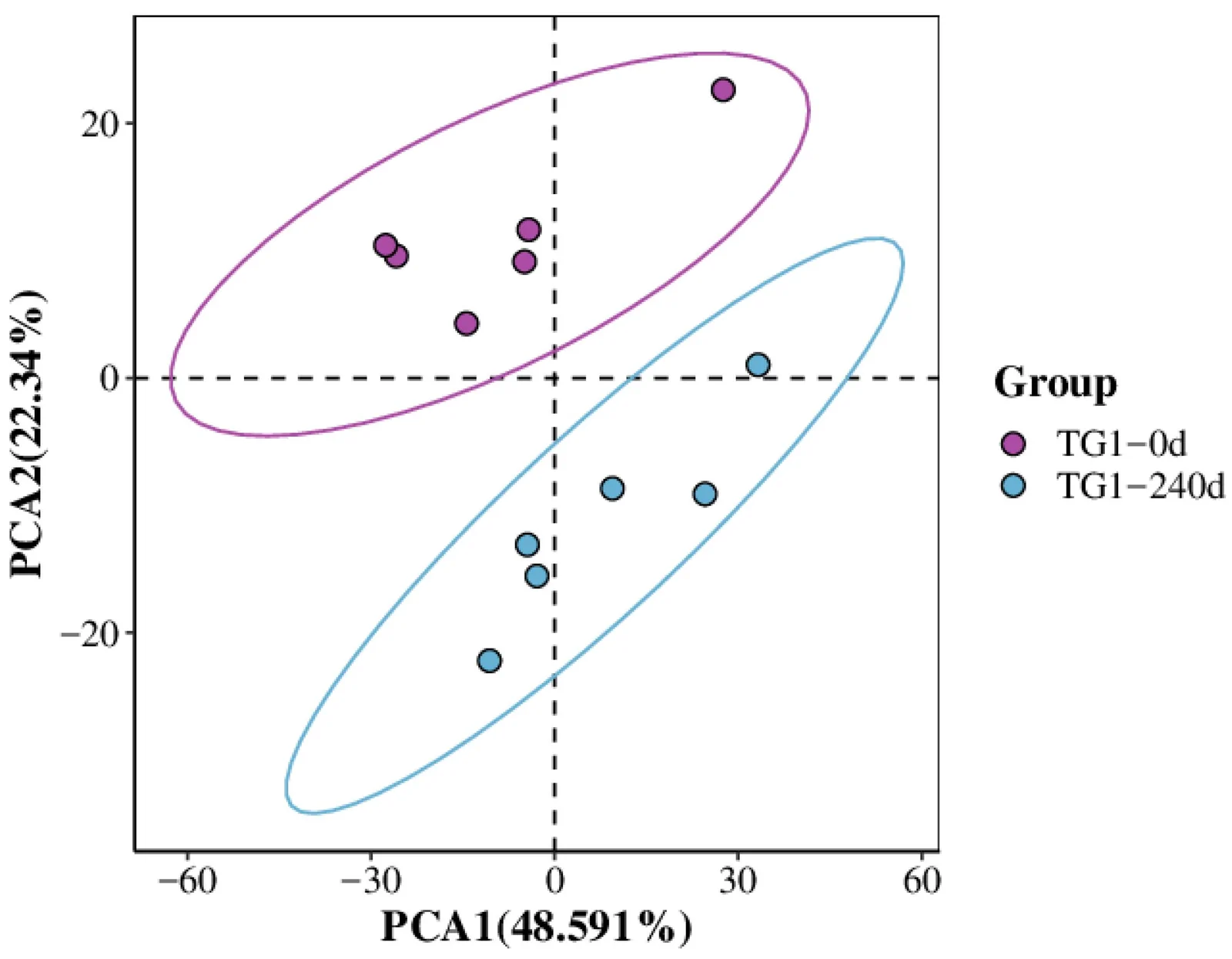
PCA score plot of TG1 before and after storage (TG1, flour supplemented with 0.2% (*w*/*w*) LBP).

**Figure 12:**
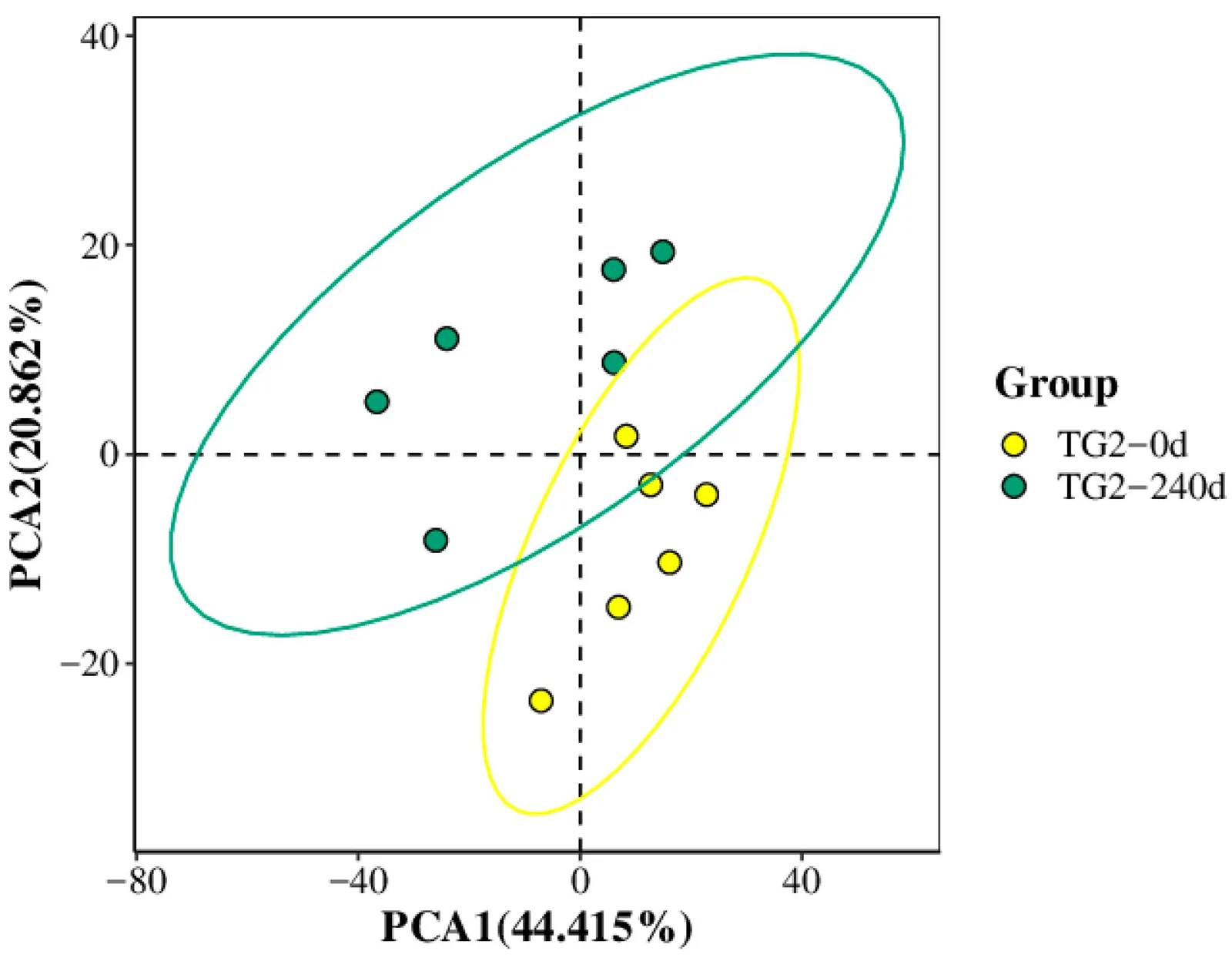
PCA score plot of TG2 before and after storage (TG2, flour supplemented with 1.0% (*w*/*w*) LBP).

**Figure 13:**
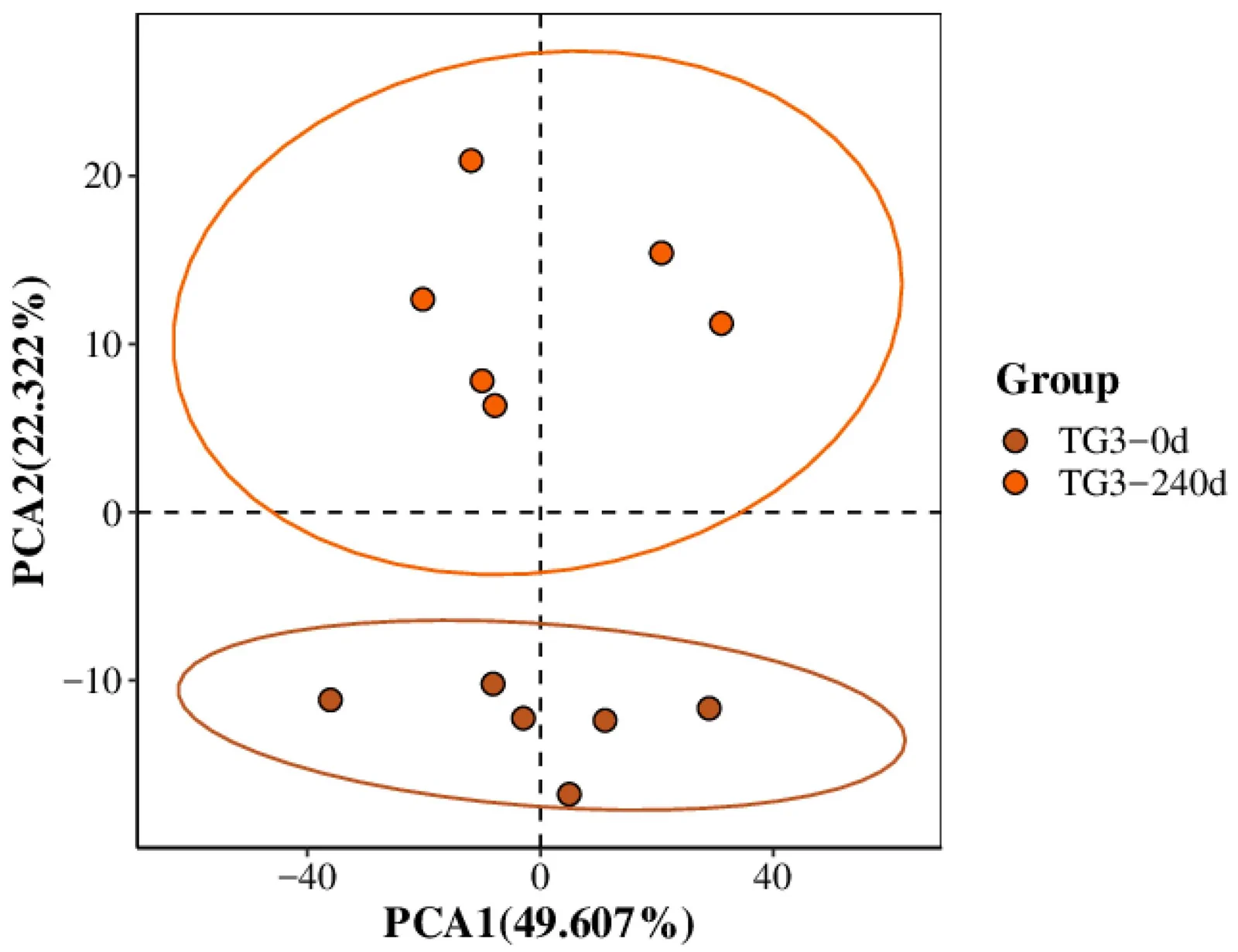
PCA score plot of TG3 before and after storage (TG3, flour supplemented with 2.0% (*w*/*w*) LBP).

A separate PCA of each group before and after storage (Figure 10, Figure 11, Figure 12 and Figure 13) showed complete separation of day-0 and day-240 samples in CK, TG1 and TG3 and near-complete separation in TG2, confirming that 240 days of storage profoundly reshaped the lipidome. OPLS-DA models for each group were robust, with R^2^Y (cum) of 0.989–0.997 and Q^2^ (cum) of 0.872–0.973 (Table 2).

#### 3.6.3. Differential Lipids

**Figure 14:**
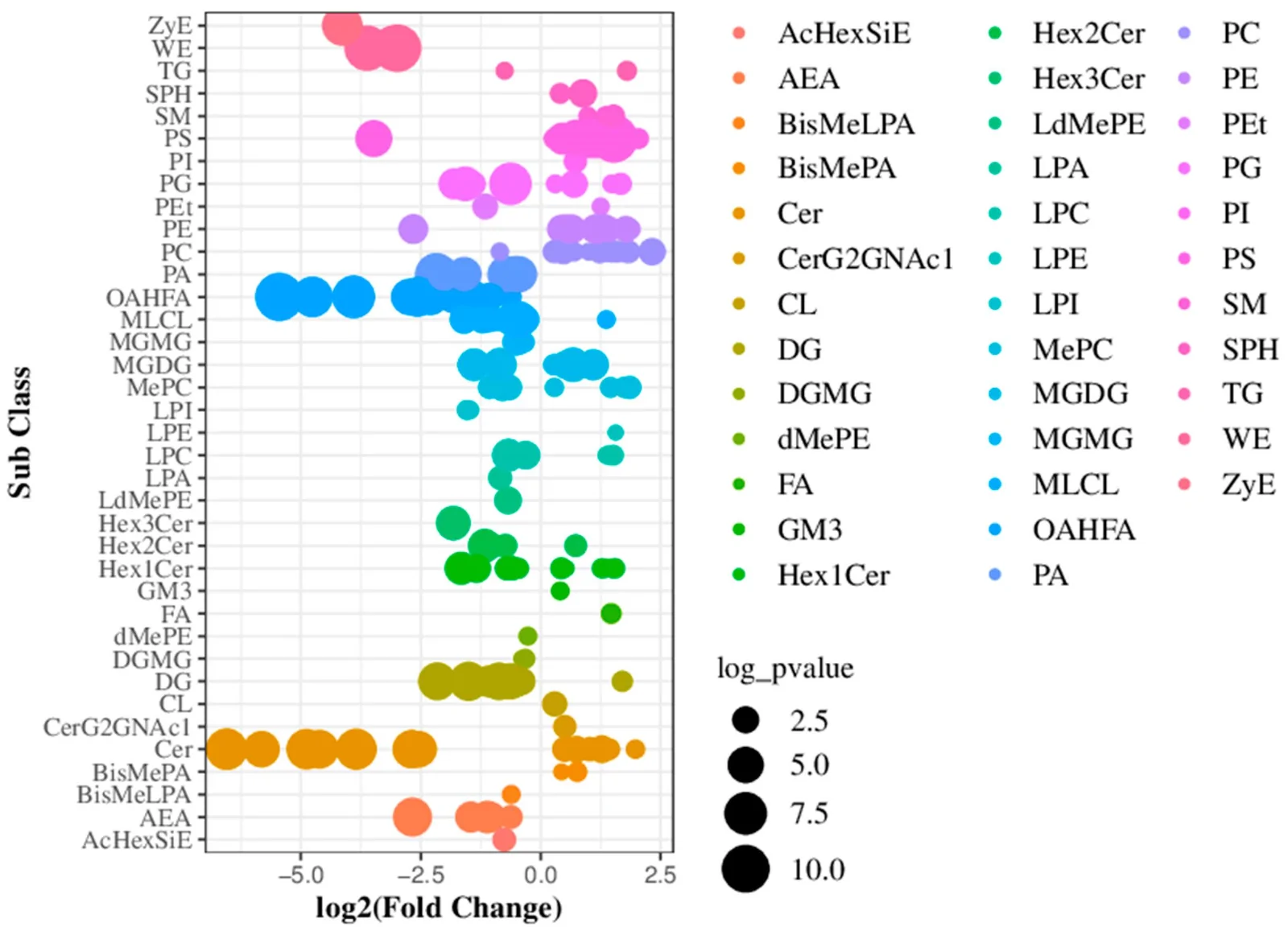
Bubble plot of the differential lipids identified in the control group (CK) before versus after 240 days of storage. The horizontal axis gives log2-transformed fold change (240 d relative to 0 d); the vertical axis lists the lipid subclasses. Each bubble represents one differential lipid, bubble colour indicates the lipid subclass to which that lipid belongs, and bubble size is proportional to the statistical significance expressed as −log10(*p*). Differential lipids were defined by VIP ≥ 1, fold change ≥1.2 or ≤0.83, and *p* < 0.05. (CK, control flour without LBP; TG1, TG2 and TG3, flour supplemented with 0.2%, 1.0% and 2.0% (*w*/*w*) LBP, respectively.)

**Figure 15:**
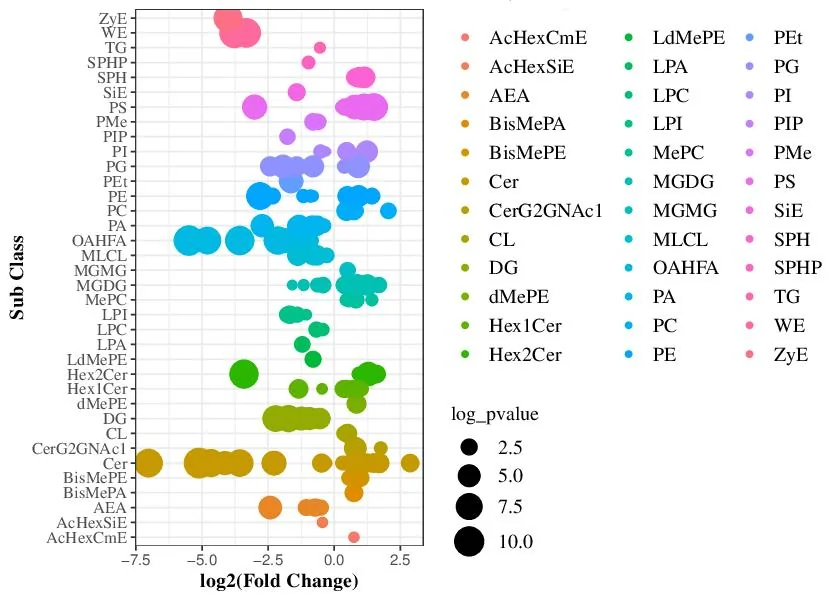
Bubble plot of differential lipids in TG1 before and after storage (TG1, flour supplemented with 0.2% (*w*/*w*) LBP).

**Figure 16:**
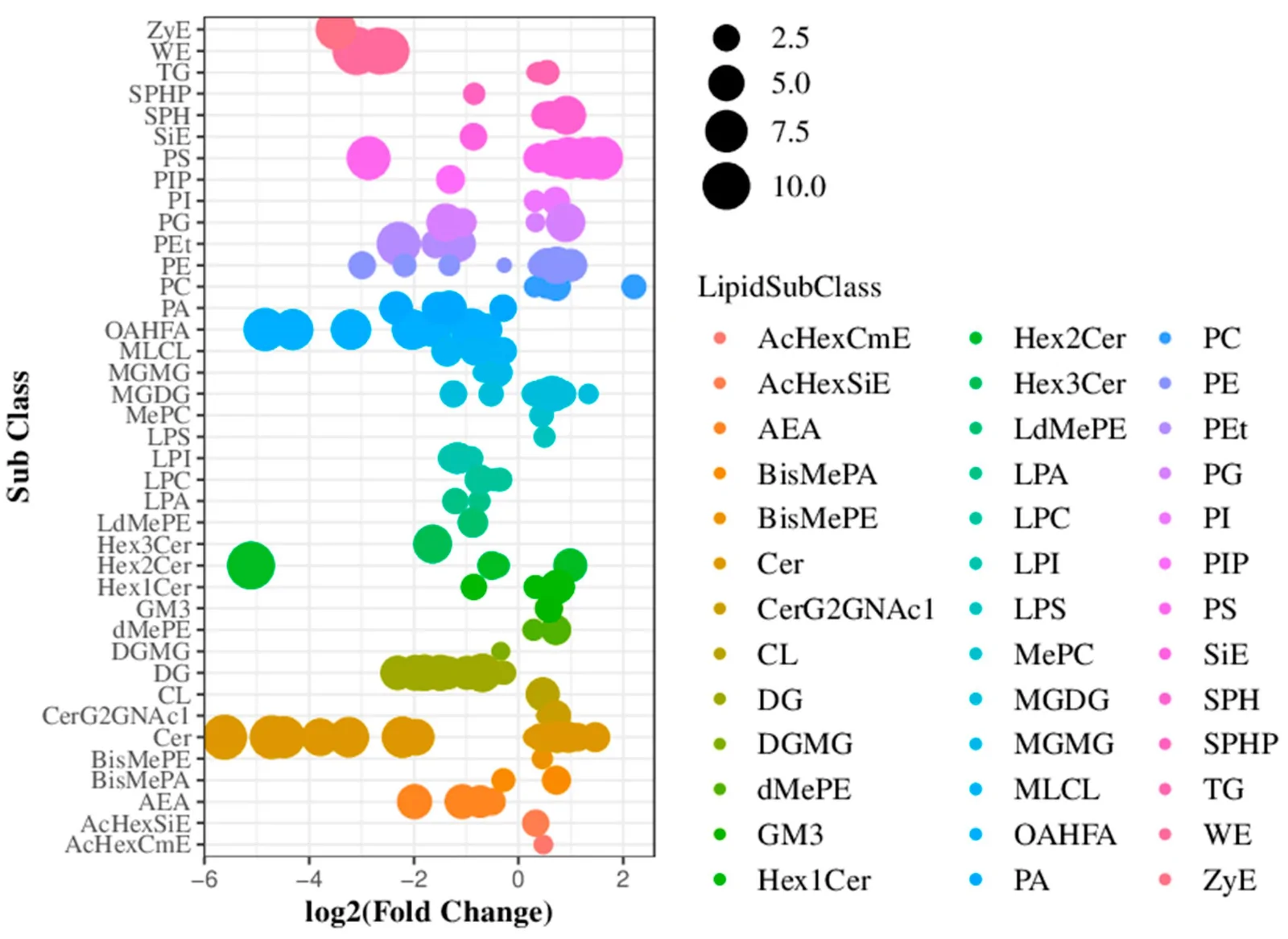
Bubble plot of differential lipids in TG2 before and after storage (TG2, flour supplemented with 1.0% (*w*/*w*) LBP).

**Figure 17:**
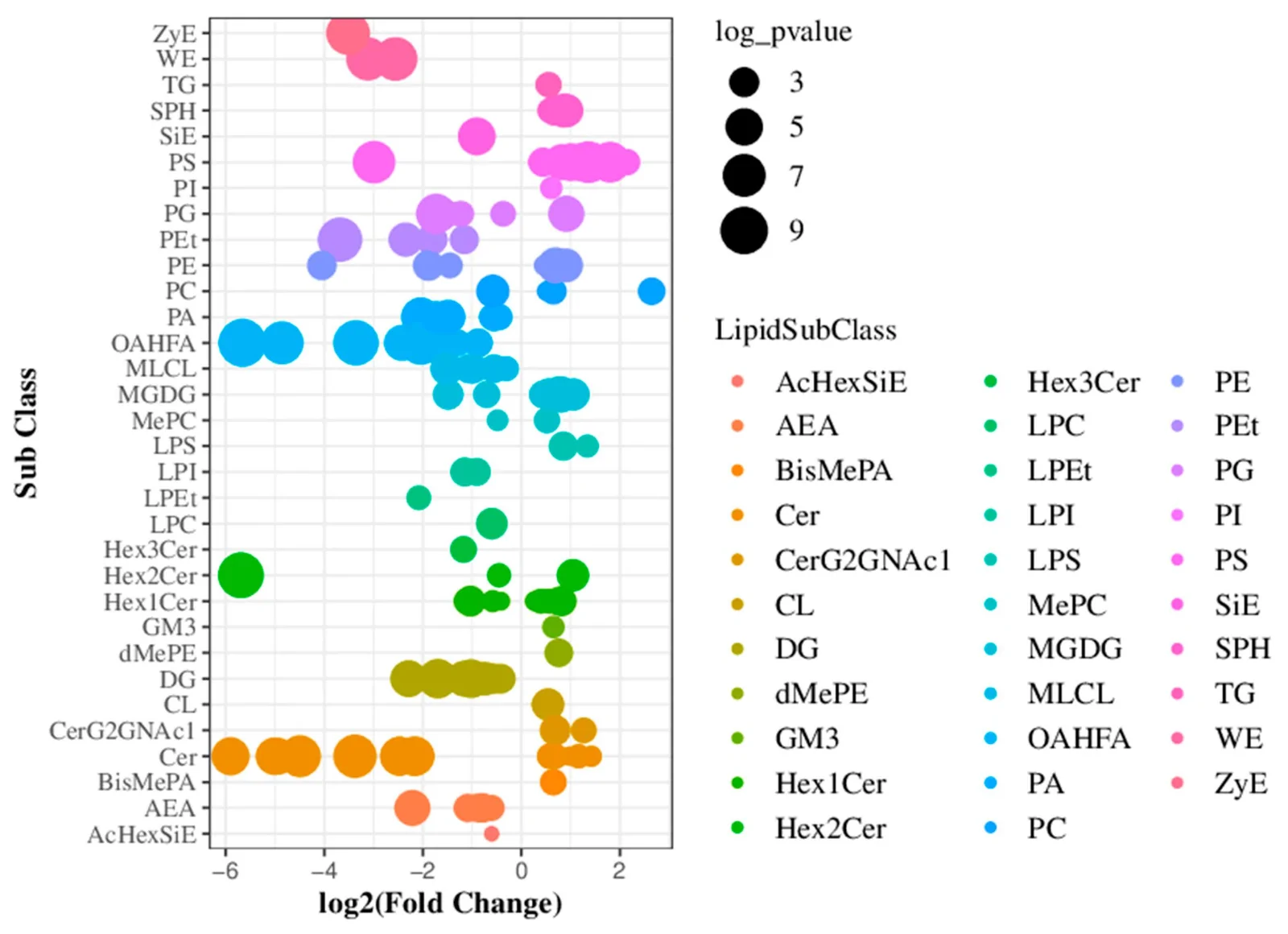
Bubble plot of differential lipids in TG3 before and after storage (TG3, flour supplemented with 2.0% (*w*/*w*) LBP).

**Figure 18:**
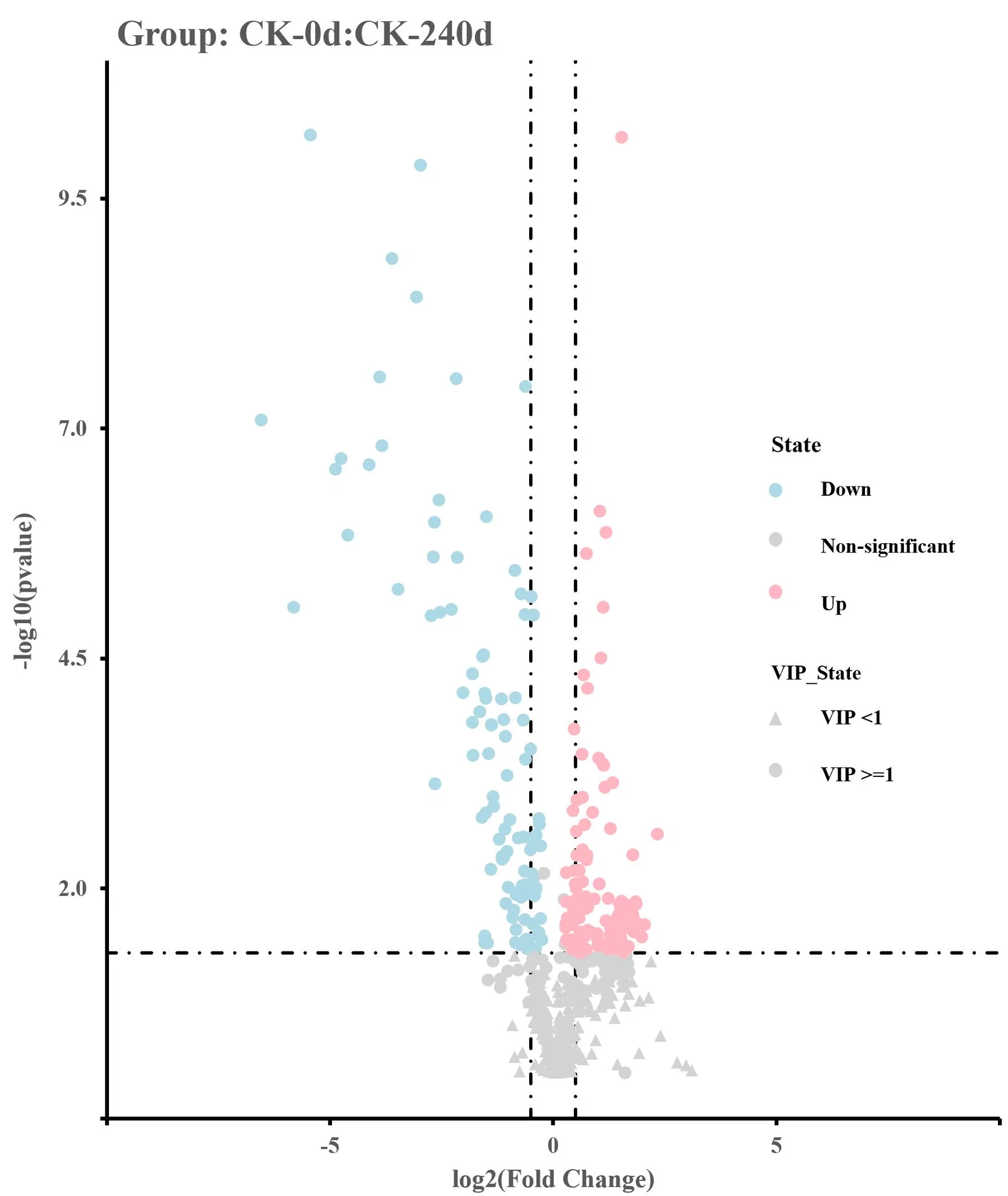
Volcano plot of the differential lipids identified in the control group (CK) before versus after 240 days of storage. The horizontal axis is log2-transformed fold change and the vertical axis is −log10(*p*). Blue symbols denote significantly downregulated lipids and red symbols significantly upregulated lipids; circles indicate lipids with VIP ≥ 1 and triangles lipids with VIP < 1; grey symbols are non-significant lipids. Differential lipids were defined by VIP ≥ 1, fold change ≥1.2 or ≤0.83, and *p* < 0.05. The axes are scaled identically in Figure 19, Figure 20, Figure 21 and Figure 22 to allow direct comparison among the treatments (CK, control flour without LBP; TG1, TG2 and TG3, flour supplemented with 0.2%, 1.0% and 2.0% (*w*/*w*) LBP, respectively).

**Figure 19:**
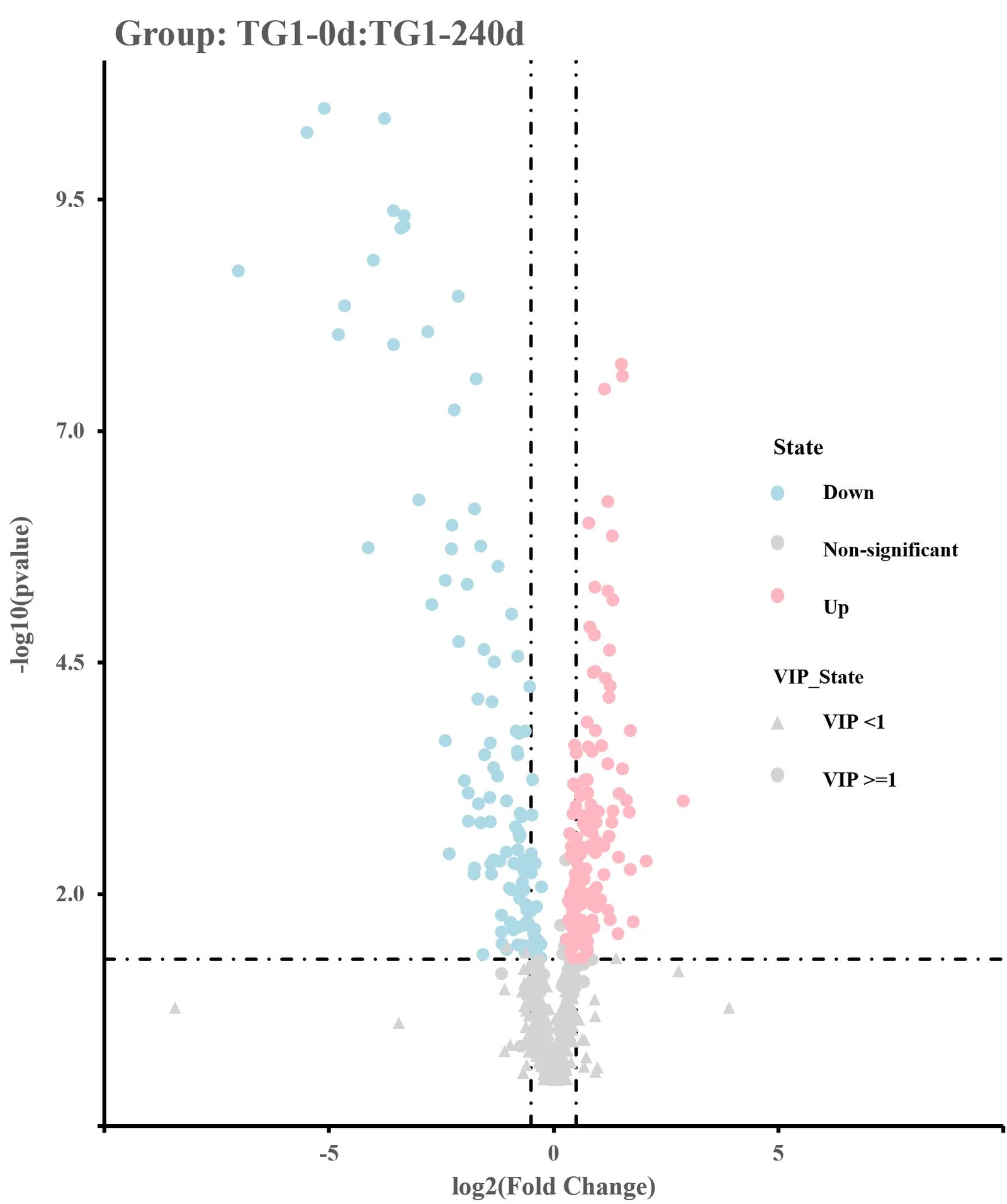
Volcano plot of differential metabolites in TG1. (TG1, flour supplemented with 0.2% (*w*/*w*) LBP).

**Figure 20:**
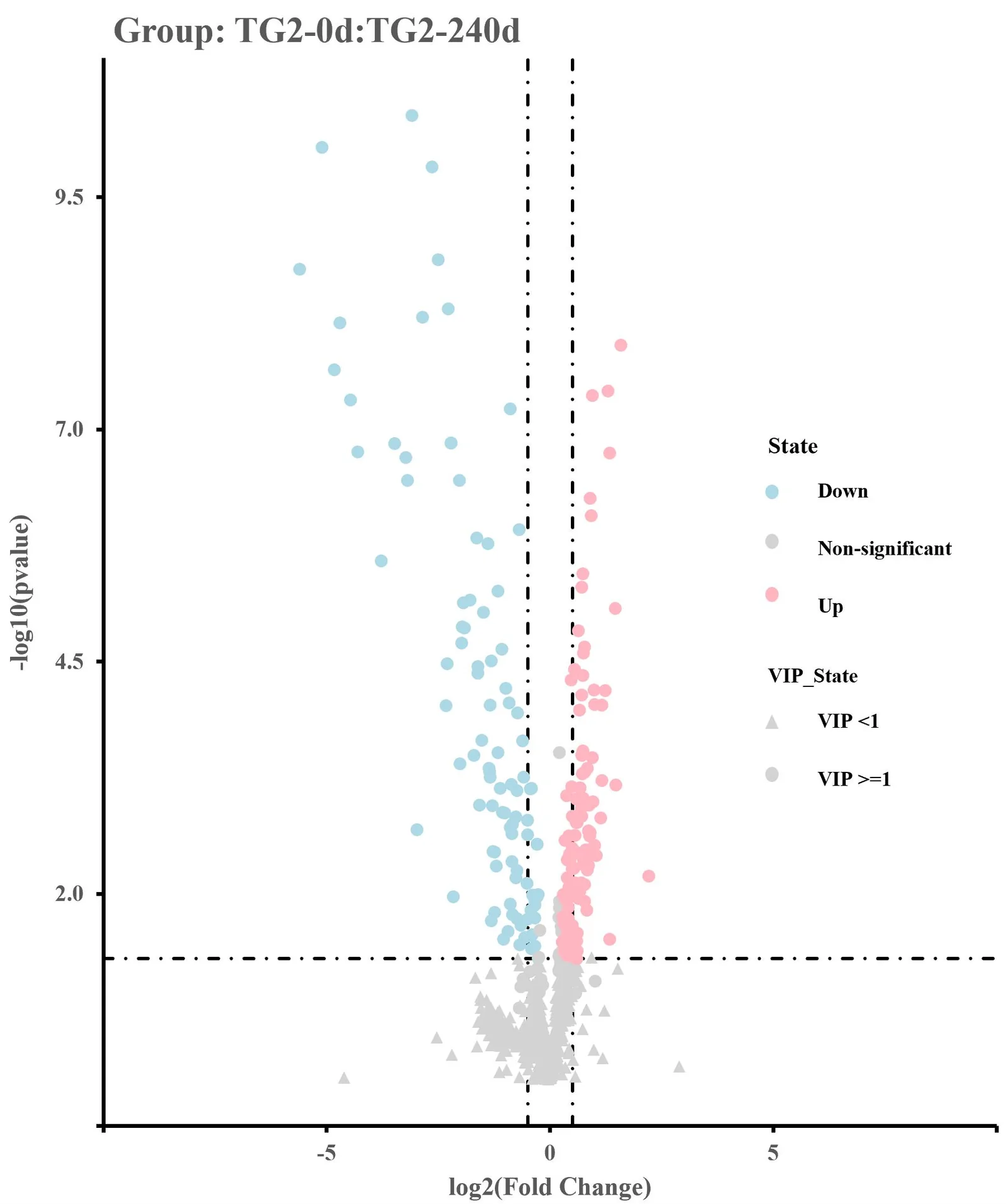
Volcano plot of differential metabolites in TG2 (TG2, flour supplemented with 1.0% (*w*/*w*) LBP).

**Figure 21:**
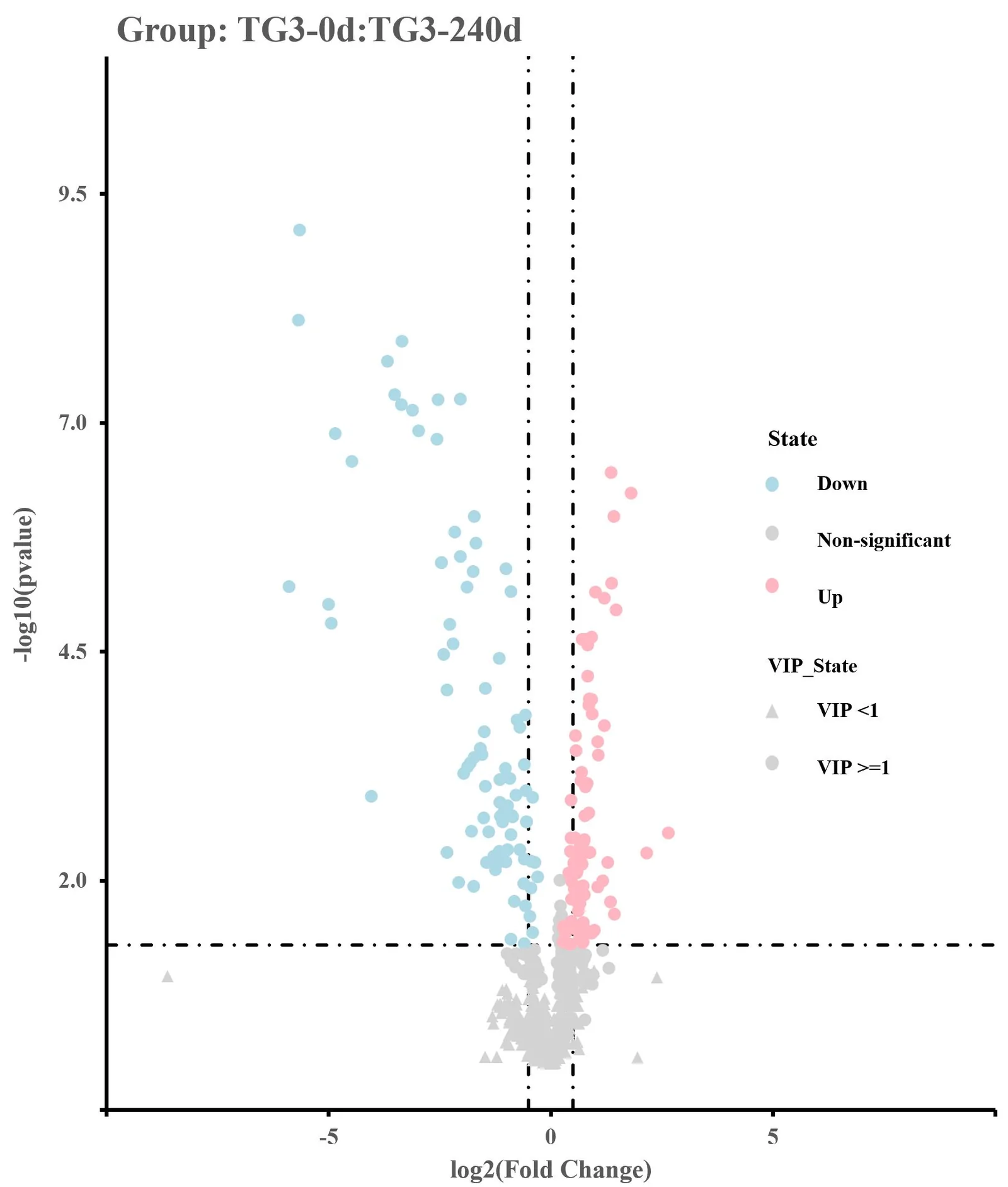
Volcano plot of differential metabolites in TG3 (TG3, flour supplemented with 2.0% (*w*/*w*) LBP).

Among the LBP-supplemented groups, the number of differential lipids decreased as the supplementation level increased: 255 in TG1, 216 in TG2 and 174 in TG3 (Table 3). The control group contained 243 differential lipids, i.e., fewer than TG1 but more than TG2 and TG3, so the dose-dependent trend applies within the LBP-treated groups and not across the full sequence from CK to TG3. The bubble plots (Figure 14, Figure 15, Figure 16 and Figure 17) showed that, relative to CK, TG1 and TG2 reduced the downregulation of membrane phospholipids (PC, PE) and storage lipids (TG) and the upregulation of lysophospholipids (LPC, LPE) and oxidized lipids, while TG3 preserved the lipid profile most completely, with minimal changes in PC, PE and MGDG and the strongest suppression of lysophospholipid formation. Volcano plots (Figure 18, Figure 19, Figure 20 and Figure 21) showed that TG1 produced the largest and most significant bidirectional changes (predominantly upregulation of lipid synthesis), whereas TG3 was the only group in which downregulated lipids outnumbered upregulated ones, indicating that 2.0% LBP shifted the metabolic direction from synthesis toward suppression of core lipid metabolism.

**Figure 22:**
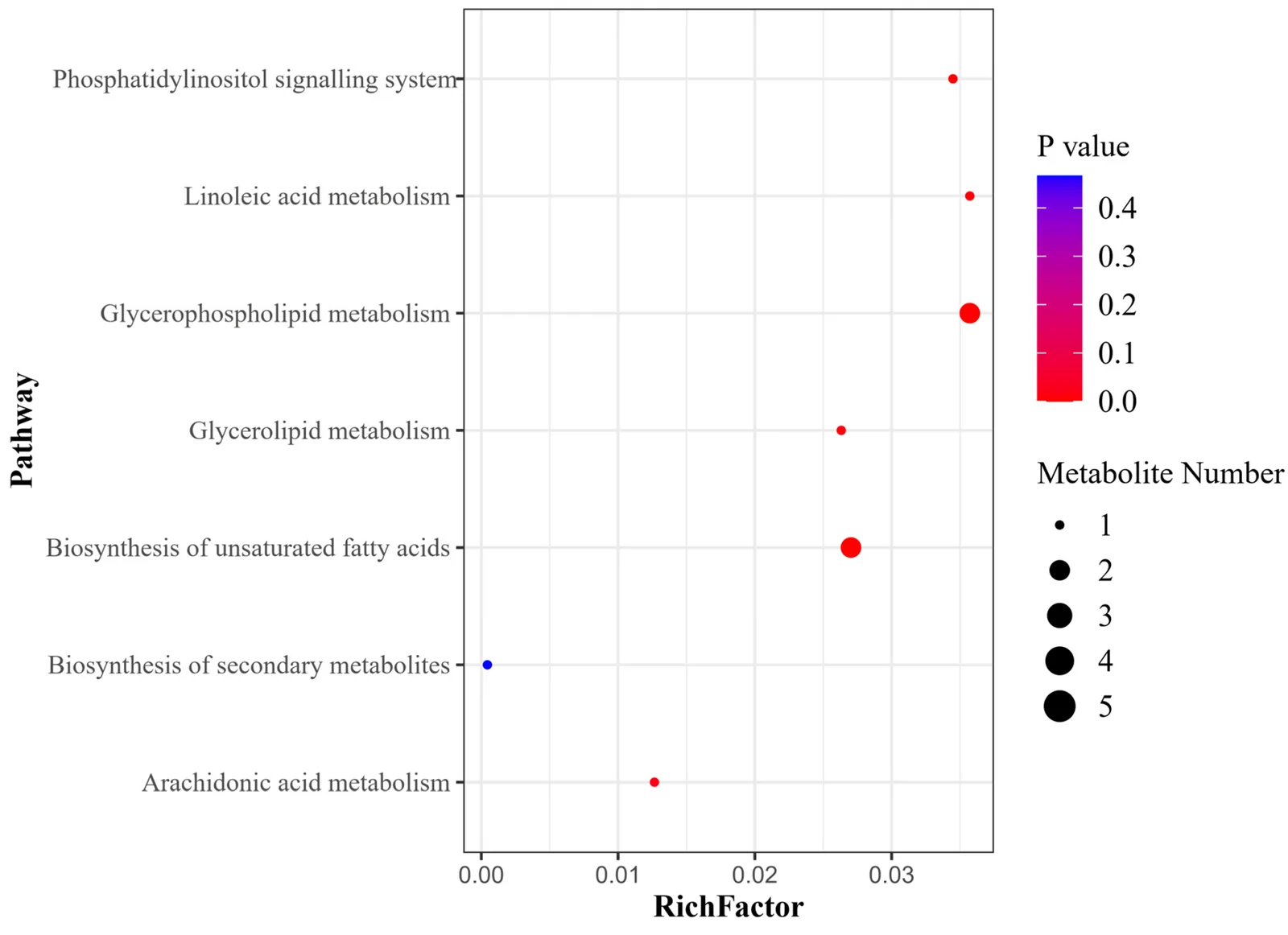
KEGG pathway enrichment analysis of the differential lipids identified in the control group (CK) before versus after 240 days of storage. The horizontal axis is the enrichment factor (RichFactor), defined as the number of differential lipids annotated to a given pathway divided by the total number of lipids annotated to that pathway; a larger value indicates that a higher proportion of the pathway’s lipids were differential. Each bubble represents one pathway, bubble size is proportional to the number of differential lipids annotated to that pathway, and bubble colour indicates the significance of the enrichment. Only pathways with *p* < 0.05 are shown; where more than ten pathways were significant, the ten with the smallest *p* values are displayed (CK, control flour without LBP; TG1, TG2 and TG3, flour supplemented with 0.2%, 1.0% and 2.0% (*w*/*w*) LBP, respectively).

#### 3.6.4. KEGG Pathway Enrichment

**Figure 23:**
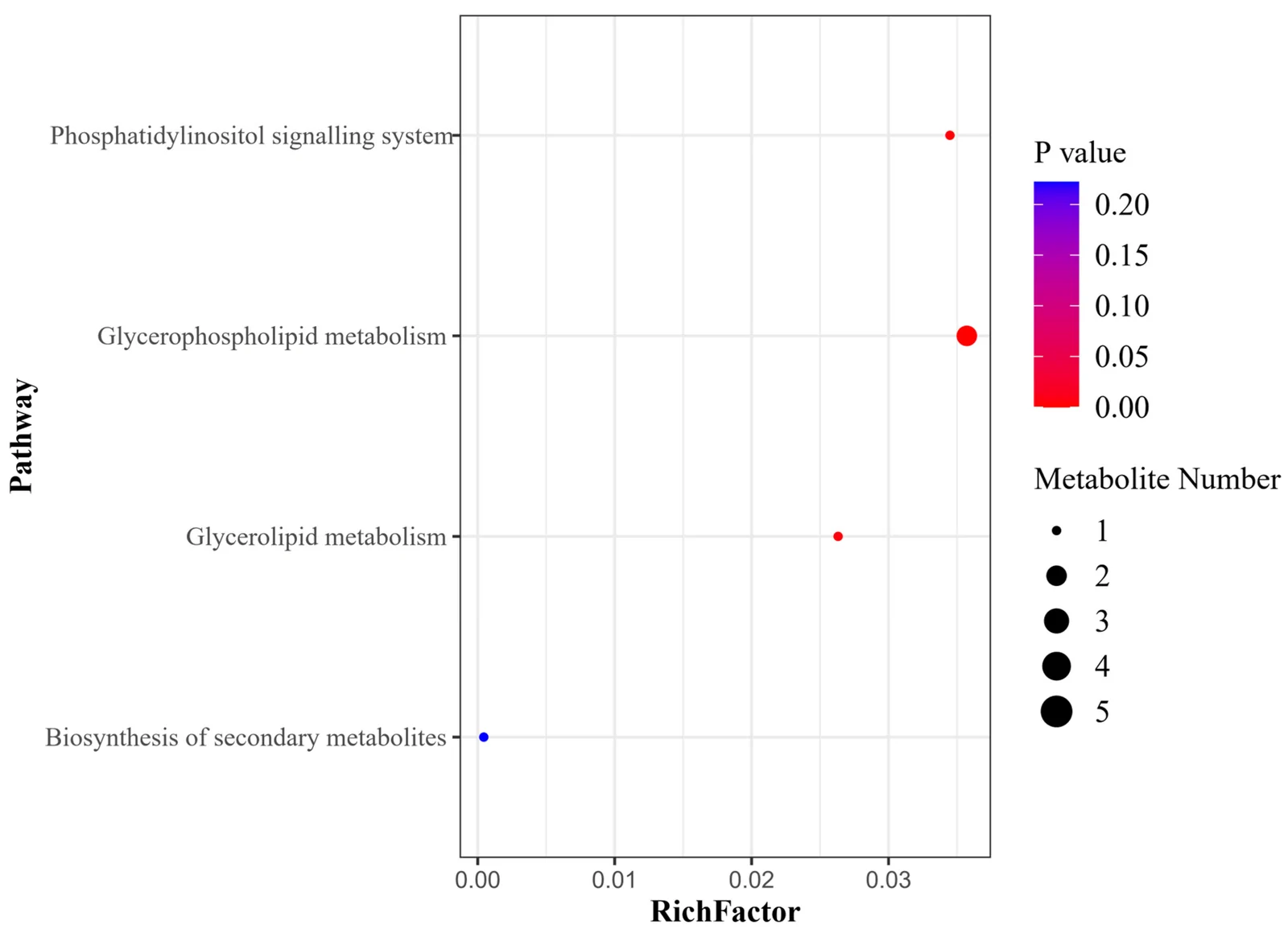
KEGG pathway enrichment bubble plot of TG1. (TG1, flour supplemented with 0.2% (*w*/*w*) LBP).

**Figure 24:**
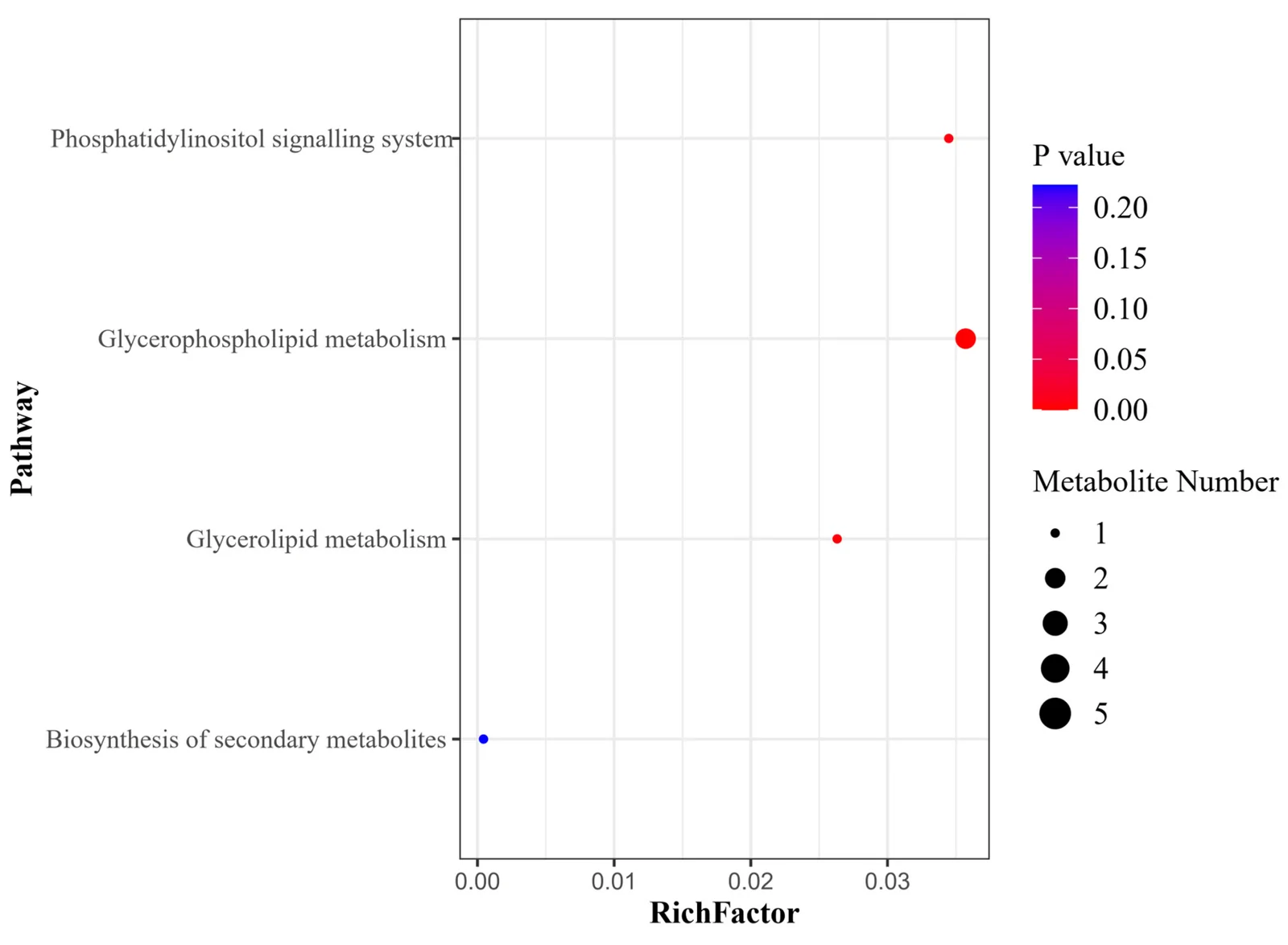
KEGG pathway enrichment bubble plot of TG2. (TG2, flour supplemented with 1.0% (*w*/*w*) LBP).

**Figure 25:**
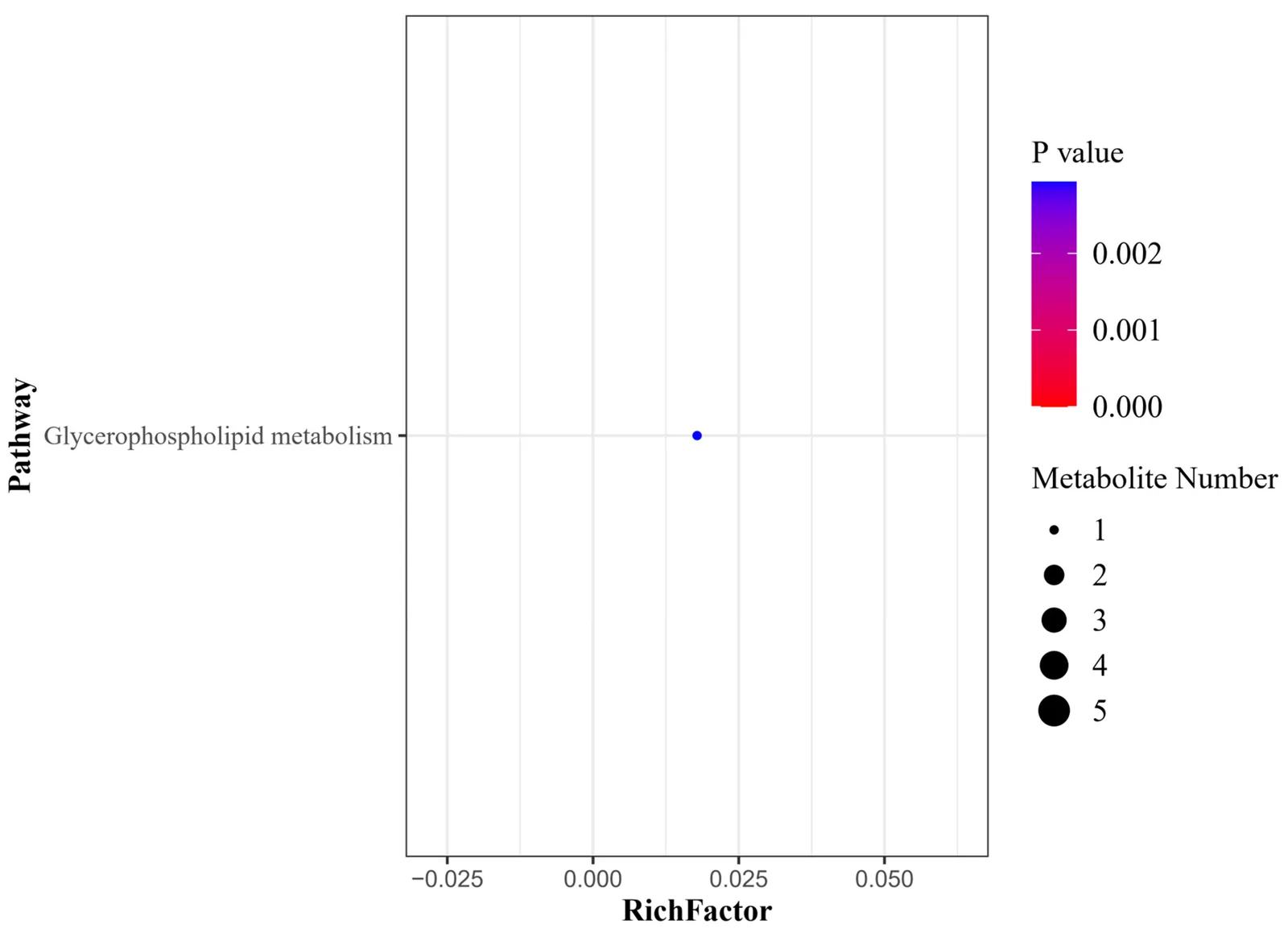
KEGG pathway enrichment bubble plot of TG3 (TG3, flour supplemented with 2.0% (*w*/*w*) LBP).

KEGG enrichment of differential lipids (Figure 22, Figure 23, Figure 24 and Figure 25) revealed seven enriched pathways in CK, of which glycerophospholipid metabolism (55.56% of annotated metabolites), glycerolipid metabolism (22.22%) and unsaturated fatty acid biosynthesis (11.11%) were significantly enriched, together with linoleic acid metabolism, arachidonic acid metabolism and the phosphatidylinositol signalling system. TG1 and TG2 each retained four enriched pathways, with glycerophospholipid metabolism, glycerolipid metabolism and the phosphatidylinositol signalling system significantly enriched, while fatty acid biosynthesis and metabolism pathways (unsaturated fatty acid biosynthesis, linoleic and arachidonic acid metabolism) lost significance. In TG3, only glycerophospholipid metabolism was detected, and it did not reach significance, showing that 2.0% LBP suppressed even the core lipid metabolic pathways.

## 4. Discussion

This study demonstrates that LBP retards the storage deterioration of highland barley flour in a dose-dependent manner and that the dose dependence differs between the macroscopic quality indices and the lipid molecular phenotype. From the perspective of integrated quality and safety, 0.2% LBP (TG1) was the most favourable level: it best preserved protein, total starch, β-glucan and GABA, kept DPPH and hydroxyl radical scavenging stable, minimized the increase in peroxide value, and most strongly suppressed total plate and mould counts. Conversely, 2.0% LBP (TG3) provided the strongest protection of the lipidome, with the fewest differential lipids and the best-preserved membrane lipids, but it also supplied a carbon source for microorganisms and suppressed the core lipid metabolic pathways. These contrasting optima highlight that the supplementation level must be selected against the intended quality target and that a moderate level (0.2%) offers the best overall benefit.

Several mechanisms can account for the protective effects of LBP. First, LBP is a hydrophilic polysaccharide whose hydroxyl-rich chains form hydrogen bonds with water molecules, slowing moisture loss. This effect became statistically detectable only at the end of the storage period (240 days) and was most pronounced at the 1.0% level; no significant differences from the control were observed at 0, 60, 120 or 180 days. Second, LBP can coat the surface of fat globules and protein bodies, reducing the contact between endogenous lipases and their substrates—consistent with the suppressed lipase activity and fatty acid value at 2.0% and with the smaller protein loss in all treated groups. Third, LBP inhibits key deteriorative enzymes, including amylases (β-glucan and starch preservation at 0.2%) [10] and polyphenol oxidase (phenolics and flavonoids preservation at 2.0%) [15]. Compared with previous studies on oat, brown rice and wheat flour stabilization, transaminase (GABA preservation at 0.2%) delays the decline in peroxidase activity, which helps clear peroxides. Fourth, LBP and its degradation products scavenge free radicals and act synergistically with endogenous phenolics and flavonoids, as reflected in the sustained ABTS scavenging activity despite declining phenolic content. Finally, LBP exerts antimicrobial activity, although at high doses the added carbohydrate can partially offset this benefit by feeding microbial growth.

The lipidomic data provide molecular-level support for these mechanisms. Within the LBP-supplemented groups, the number of differential lipids decreased as the supplementation level increased (255 → 216 → 174 for 0.2%, 1.0% and 2.0% LBP, respectively; the control group contained 243), and the progressively smaller changes in PC, PE, MGDG and TG indicate that LBP stabilizes both membrane and storage lipids, while the suppressed accumulation of LPC and LPE shows that phospholipid hydrolysis—the earliest molecular signature of membrane damage—is inhibited. This is consistent with the conventional indices: a lower fatty acid value and MDA content in treated groups indicate slower TAG hydrolysis and lipid peroxidation, and the lipidomic picture explains why these macroscopic markers improved. The KEGG results add an important nuance: 0.2–1.0% LBP maintained the activity of the core glycerophospholipid and glycerolipid synthesis pathways while silencing fatty acid biosynthesis pathways, suggesting that low-to-moderate doses act on downstream lipid remodelling rather than on de novo fatty acid synthesis; 2.0% LBP went further and suppressed even the core pathways. The near-complete silencing at 2.0% may reflect the markedly different metabolic direction of TG3 (net downregulation) and could be related to the microbial carbon-source effect at high doses, which diverts available nutrients toward microbial consumption rather than lipid synthesis.

Compared with previous studies on oat, brown rice and wheat flour stabilization [16,17], the present work adds a dose–response dimension that is rarely examined: most studies evaluate a single addition level and cannot distinguish whether the protective effect saturates or reverses. The combination of a 240-day real-time storage trial with untargeted lipidomics further links the macroscopic quality trajectory to the molecular lipid phenotype, an integration that is uncommon in the cereal flour preservation literature. From an application standpoint, the results indicate that 0.2% (*w*/*w*) LBP was the most effective of the levels tested in preserving the overall quality of highland barley flour during storage. Because the present study included neither an economic assessment nor a dedicated safety evaluation of LBP (e.g., toxicological testing or residue analysis), no conclusion on the cost-effectiveness or safety of LBP can be drawn here, and both aspects should be assessed before application.

Several limitations should be acknowledged. First, only one cultivar (Kunlun-15) and one batch of LBP were tested; the LBP was a laboratory-prepared preparation of defined total polysaccharide content (90%, *w*/*w*) but was not further characterized with respect to its molecular weight distribution or monosaccharide composition, and the optimal dose may vary with cultivar, milling degree and polysaccharide composition. Second, storage was conducted under a single ambient regime (ca. 11 °C, 45% RH); accelerated conditions could change the dominant deterioration pathway. Third, the four groups were compared within each storage time by one-way ANOVA, so the interaction between LBP level and storage time was not statistically tested; the correlation analyses identify associations rather than causal links; the differential-lipid and pathway-enrichment criteria used unadjusted *p* values without multiple-testing correction, so the reported lipid sets should be regarded as candidates for further verification; and the KEGG annotations rely on plant-generic pathway databases that may not fully reflect barley-specific metabolism. Fourth, microbial community composition was not profiled, although the total counts indicate clear treatment effects. Future work should examine multiple cultivars and storage regimes, profile the microbial community by amplicon sequencing, and verify the causal role of specific enzymes (e.g., lipase, polyphenol oxidase) using purified enzyme assays.

## 5. Conclusions

*Lycium barbarum* polysaccharide delays the storage deterioration of highland barley flour, with the most favourable effects differing among the supplementation levels tested. Among these levels, 0.2% (*w*/*w*) LBP performed best overall, preserving protein, total starch, β-glucan and GABA, stabilizing antioxidant activity, suppressing microbial growth and slowing lipid oxidation, and is therefore recommended as the most suitable of the levels tested. Untargeted lipidomics revealed the underlying molecular mechanism: LBP protects membrane phospholipids (PC, PE), storage lipids (TG) and galactolipids (MGDG), suppresses the formation of lysophospholipids (LPC, LPE), and reduces the number of differential lipids as the supplementation level increases within the LBP-treated groups (255 → 216 → 174), while moderate doses (0.2–1.0%) preserve the core glycerophospholipid and glycerolipid metabolic pathways. These findings support the use of LBP as a natural preservative for whole-grain flours and provide a lipid-level rationale for dose selection in cereal flour stabilization.

## Figures and Tables

**Table 1:** Baseline values of the basic nutritional and functional components of highland barley flour in the four groups at day 0 (mean ± SD, *n* = 3). The day-0 values are the reference for evaluating all subsequent storage changes.

Index	CK	TG1 (0.2%)	TG2 (1.0%)	TG3 (2.0%)
Moisture (%)	10.36 ± 0.05 ^a^	10.26 ± 0.07 ^a^	10.23 ± 0.06 ^a^	10.26 ± 0.03 ^a^
Crude fat (%)	3.81 ± 0.53 ^a^	3.54 ± 0.21 ^a^	3.80 ± 0.13 ^a^	3.82 ± 0.13 ^a^
Protein (%)	9.32 ± 0.26 ^a^	9.17 ± 0.21 ^a^	9.21 ± 0.26 ^a^	9.33 ± 0.26 ^a^
Total starch (%)	55.41 ± 2.06 ^a^	56.45 ± 1.19 ^a^	59.04 ± 3.24 ^a^	54.06 ± 4.56 ^a^
β-Glucan (g/100 g)	6.70 ± 0.10 ^a^	6.81 ± 0.19 ^a^	6.57 ± 0.22 ^a^	7.05 ± 0.07 ^a^
Total phenolics (mg/g)	6.34 ± 0.05 ^a^	6.46 ± 0.06 ^a^	6.55 ± 0.02 ^a^	6.59 ± 0.23 ^a^
Total flavonoids (mg/g)	2.39 ± 0.03 ^a^	2.45 ± 0.04 ^a^	2.33 ± 0.02 ^a^	2.37 ± 0.02 ^a^
GABA (mg/100 g)	10.20 ± 0.14 ^a^	10.36 ± 0.19 ^a^	10.19 ± 0.08 ^a^	10.35 ± 0.09 ^a^
Lipase activity (mg/g)	7.47 ± 0.31 ^a^	7.38 ± 0.35 ^a^	7.16 ± 0.51 ^a^	6.97 ± 0.20 ^a^
Fatty acid value (mg/100 g)	22.00 ± 2.26 ^a^	18.01 ± 1.23 ^a^	18.76 ± 1.22 ^a^	20.46 ± 2.47 ^a^
MDA (nmol/g)	7.90 ± 0.21 ^a^	7.53 ± 0.04 ^a^	7.24 ± 0.06 ^a^	7.81 ± 0.94 ^a^
Peroxide value (meq/kg)	1.71 ± 0.01 ^a^	1.70 ± 0.04 ^a^	1.69 ± 0.01 ^a^	1.69 ± 0.01 ^a^
Peroxidase activity (U/g·min)	376.75 ± 1.68 ^a^	370.24 ± 2.15 ^a^	367.81 ± 1.07 ^a^	365.77 ± 3.39 ^a^
Total plate count (×10^3^ CFU/g)	3.22 ± 0.03 ^a^	3.15 ± 0.02 ^a^	3.17 ± 0.03 ^a^	3.19 ± 0.02 ^a^
Mold count (CFU/g)	43.00 ± 1.00 ^a^	39.00 ± 2.00 ^a^	41.00 ± 1.00 ^a^	42.00 ± 1.00 ^a^

Within each row, values sharing the same lowercase letter do not differ significantly among the four groups at day 0 (Duncan’s multiple-range test, *p* < 0.05).

**Table 2:** Evaluation parameters of the OPLS-DA models for each group (before vs. after 240-day storage).

Group	R^2^Y (cum)	Q^2^ (cum)
CK (0 d vs. 240 d)	0.997	0.954
TG1 (0 d vs. 240 d)	0.989	0.955
TG2 (0 d vs. 240 d)	0.997	0.872
TG3 (0 d vs. 240 d)	0.997	0.973

**Table 3:** Number of differential lipids (fold change ≥1.2) identified in each group before vs. after 240-day storage.

Group	Differential Lipids (Total)
CK (0 d vs. 240 d)	243
TG1 (0 d vs. 240 d)	255
TG2 (0 d vs. 240 d)	216
TG3 (0 d vs. 240 d)	174

## Data Availability

The original contributions presented in this study are included in the article. Further inquiries can be directed to the corresponding authors.
